# Growth Prediction in Orthodontics: ASystematic Review of Past Methods up to Artificial Intelligence

**DOI:** 10.3390/children12081023

**Published:** 2025-08-03

**Authors:** Ioannis Lyros, Heleni Vastardis, Ioannis A. Tsolakis, Georgia Kotantoula, Theodoros Lykogeorgos, Apostolos I. Tsolakis

**Affiliations:** 1 Department of Orthodontics, School of Dentistry, National and Kapodistrian University of Athens, 11527 Athens, Greece; vastard@dent.uoa.gr (H.V.); geokotantouladds@yahoo.com (G.K.); apostso@otenet.gr (A.I.T.); 2 Department of Orthodontics, School of Dentistry, Aristotle University of Thessaloniki, 54623 Thessaloniki, Greece; tsolakisioannis@gmail.com; 3 Department of Orthodontics, Case Western Reserve University, Cleveland, OH 44106, USA; 4 “Hatzikosta” General Hospital of Messolonghi, 30200 Messolonghi, Greece; theolyk@gmail.com

**Keywords:** artificial intelligence, orthodontic treatment, growth prediction, cephalometric radiography, hand–wrist radiography, cervical vertebrae, metal implants, deep learning, machine learning

## Abstract

**Highlights:**

**Main findings:**

- This systematic review presents both traditional and concurrent digital methods for predicting human growth. A systematic search identified 69 studies on maxillofacial growth prediction lacking any orthodontic intervention.
- Skeletal age is commonly assessed using cervical vertebrae and hand–wrist radiographs. Alternative approaches, including metal implants, biochemical markers, and electromyography, have been implemented.

**Implications of the main findings:**

- Emerging digital tools aim to enhance the accuracy of traditional methods.
- Future growth prediction should aim to minimize patient distress and radiation exposure. A more comprehensive and reliable prediction model may emerge from integrating established techniques with AI and digital innovations.

**Abstract:**

Background/Objectives: Growth prediction may be used by the clinical orthodontist in growing individuals for diagnostic purposes and for treatment planning. This process appraises chronological age and determines the degree of skeletal maturity to calculate residual growth. In developmental deviations, overlooking such diagnostic details might culminate in erroneous conclusions, unstable outcomes, recurrence, and treatment failure. The present review aims to systematically present and explain the available means for predicting growth in humans. Traditional, long-known, popular methods are discussed, and modern digital applications are described. Materials and methods: A search on PubMed and the gray literature up to May 2025 produced 69 eligible studies on future maxillofacial growth prediction without any orthodontic intervention. Results: Substantial variability exists in the studies on growth prediction. In young orthodontic patients, the study of the lateral cephalometric radiography and the subsequent calculation of planes and angles remain questionable for diagnosis and treatment planning. Skeletal age assessment is readily accomplished with X-rays of the cervical vertebrae and the hand–wrist region. Computer software is being implemented to improve the reliability of classic methodologies. Metal implants have been used in seminal growth studies. Biochemical methods and electromyography have been suggested for clinical prediction and for research purposes. Conclusions: In young patients, it would be of importance to reach conclusions on future growth with minimal distress to the individual and, also, reduced exposure to ionizing radiation. Nevertheless, the potential for comprehensive prediction is still largely lacking. It could be accomplished in the future by combining established methods with digital technology.

## 1. Introduction

From birth up to adulthood, growth and development involve incessant dimensional and functional changes [1,2,3]. Actually, tissue growth refers to a volumetric or weight increase and is connected with alterations occurring throughout the lifetime. Importantly, human biological cycle comprises both growth and maturation [4]. Namely, procedures of the maturing body can be traced in the growing craniofacial complex [5,6,7]. The orthodontist should be concerned with occlusal stability and facial appearance as they are associated with self-image, social, and psychological well-being [8,9,10]. For a clinician planning to achieve desired facial features, external manifestations should be related to the underlying osseous foundation providing adequate support [11,12,13,14]. Facial growth studies shed light on specific regions of remodeling and growth, disclosing the contributing tissues [15,16,17]. In particular, any treatment that involves functional appliances and surgical intervention is closely connected with assessing and predicting the growth potential of the individual [18,19,20,21,22]. Quite often, the orthodontist makes predictions on the young patient’s facial dimensions with reference to the parents and siblings, as similarities within families are not uncommon [23,24,25]. Suzuki and Takahama (1991) demonstrated that the craniofacial dimensions of children tend to resemble their parents’ throughout the period of growth. Therefore, they commented it is better to use such parental information to determine future outcomes than resort to average growth curves [26].

As early as in the 18th century, a longitudinal growth study was conducted, albeit inadvertently, by Philibert Guéneau de Montbeillard, a lawyer, writer, and naturalist, advocate of inoculation against smallpox, involving his son, François, from 1759 to 1777, to be later published in “Histoire Naturelle” by French Georges-Louis Leclerc, Comte de Buffon, a well-known naturalist himself. In 1927, Professor of Anatomy Richard E. Scammon presented the findings in a brief article under the title “The First Seriatum Study of Human Growth”, which was published in the *American Journal of Physical Anthropology,* although such information on human growth was already known. The French boy’s growth, measured nearly biannually, was plotted graphically in full, from birth up to adulthood. Scammon’s report boosted interest in ensuing growth studies throughout the 1930s [27].

In younger orthodontic patients, predicting residual growth is of great interest as it contributes to the understanding and the study of an emerging malocclusion. Moreover, when planning maxillofacial functional and esthetic improvement, it helps determine the optimal timing for intervention [28,29,30]. The skeletal age is considered the most reliable biological marker of maturation, not necessarily closely related to the chronological age [31,32,33] or the individual’s growth curve [34,35,36,37]. Allegedly, the average growth curves exhibit such variability that they might prove irrelevant for the individualized diagnosis of developmental deviations or for growth prediction. Each individual matures on a specific schedule [19,38]. According to Mellion et al. (2013), there is a more consistent association between facial growth and skeletal maturity around the timing of the male pubertal growth spurt [39].

For any method of prediction to be regarded ethically and practically acceptable, it ought to involve a straightforward procedure of testing, minimal exposure to radiation [40], enough accuracy to suggest appropriate treatment, and also high acceptability from predominantly young patients [32,41]. Furthermore, it should have been modeled for growth forecasts based on observations at an early age, namely, to identify outcomes occurring in the period following puberty on the basis of prepubescent data. In addition, it must refer to both genders and to differing patterns of growth [42,43]. The utility of any methodology should be appraised on its performance in a sample featuring average population values [44].

In clinical practice, to estimate the level and rate of maturation may necessitate details on dental age [45], skeletal age [46,47], the gender and accompanying secondary sex features, the body type [48,49], and genetic factors [37,50,51]. Sadly, dental age is loosely and inconsistently correlated with general growth [52,53,54]. Therefore, estimating the skeletal age may prove more reliable and thus could be readily used for developmental prediction [36,55]. Methods based on skeletal age include the measurement of ossification, the probability-based prediction, the percentage increase, the multiple regression method, and the implementation of a diagram [56]. Recording growth serves to understand evolutionary pathways affecting morphological variance, often produced by differing growth rates of various components of the musculoskeletal body structure [57].

Currently, the study of head and face osseous growth, of particular interest in orthodontics, is mainly accomplished with the aid of cephalometric radiographic imaging. In children, cervical vertebral X-rays are appraised [58], and may be supplemented with distal hand images at varying ages [26]. In addition, methods such as the introduction of metal implants [59,60,61,62], biochemical testing in the context of tissue culture [63], radioisotope administration [64,65] and electromyography (EMG) [66,67,68] have been implemented in growth studies.

Artificial intelligence (AI) is the technology that enables machines to “become intelligent”, to mimic human brain function with the aid of smart algorithms, namely computer programs. AI makes computers replicate human intelligence without restrictions due to biology [69]. Implementing AI in orthodontics to predict future growth may improve accuracy and consistency. It could also enhance time and financial efficiency [70].

Machine learning (ML), a section of AI featuring a more open-sourced code, predicts outcomes capitalizing on algorithms and data. It is highly adaptable, capable of generalizing and processing large-scale data. Its main goal is to enable computers to learn from existing data how to solve problems with minimal human intervention. By analyzing patterns, ML systems can reach decisions and self-improve over time [71]. In the supervised learning technique, training data and human feedback are used to “teach” an algorithm the relationship between inputs and outputs [72]. After analyzing the data to identify the patterns, the system can proceed to more reliable and accurate predictions on novel issues [73]. Unsupervised learning means that the training of the models is based on unknown datasets, the procedure running without supervision [74].

Artificial neurons are used by neural networks (NNs), a special kind of algorithm, to process data. The main objective of NN introduction is the creation of systems capable of tasks that imitate the functioning of the human brain [75]. The networks use data analysis and ML to make judgments and solve problems. In many different applications, NNs are utilized to simulate the processing and comprehension of the brain [70]. The fundamental components of artificial neural networks (ANNs), a sub-domain of machine learning [76], are the node layers, which include an input layer, an output layer, and one or more hidden layers that interconnect. ANNs have been inspired by the human brain’s biological neural system [76]. Biological and network neurons are comparable. They receive information from the other neurons, processit and then produce a result. NNs require training data to learn and become progressively more accurate. After adjusting for precision, these learning algorithms become useful instruments for swift data grouping [77,78]. ANNs with multiple hidden layers and advanced algorithms are commonly called as deep learning (DL) [79] and perform well in tasks such as classification and segmentation [80,81,82]. In addition, DL allows for automated feature extraction without the operator intervening, and thus, the necessary information can be accessed more predictably within a large volume of data [83]. Convolutional neural networks (CNNs), a widely used DL algorithm, perform remarkably well in image manipulation [84].

AI is being used in orthodontics for facial type and growth prediction. Automating cephalometric analysis has been introduced in an attempt to decrease the amount of time needed for analysis acquisition, enhance the precision of landmark recognition, and decrease errors stemming from clinicians’ subjectivity. A digital cephalometric radiography must be loaded on the computer; the program locates the landmarks and automatically calculates measurements needed for the analysis [85]. For instance, a fraction of AI-empowered, commercially available online applications isCephX, WebCeph^©^, Dolphin Imaging^©^, Quick Ceph^©^, and AudaxCeph^©^ [86].

The present review aspires to discuss and systematically appraise proposed ways of growth prediction, explaining their application in the clinical setting and in research. The significance of the procedure becomes evident by the current worldwide research endeavor that increasingly benefits from computer technology and up-to-date AI networks [87,88]. The qualified orthodontist is properly trained to select the appropriate tool for the reliable prediction of future growth, the planning of facial estheticimprovement, and the adjustment of oral functioning. Thus, the ensuing treatment outcome is likely to prove more stable and pleasing in the long term [18].

## 2. Materials and Methods

A detailed protocol was created and tested based on the PRISMA-P statement guidelines [89]. The process adhered to the recommendations outlined in the *Cochrane Handbook for Systematic Reviews of Interventions* [90] and the PRISMA statement [91]. The article was registered in Open Science Forum Database following the Prisma-P guidelines and received the Protocol: 10.17605/OSF.IO/BVU2M.

### 2.1. Eligibility Criteria

Eligibility criteria were established using the PICOS framework (Participants, Intervention, Comparison, Outcomes, and Study design) as outlined in Table 1. Included studies focused on healthy individuals or their radiographic images. Review articles and meta-analyses were excluded from consideration.

### 2.2. Information Sources and Search Strategy

The PubMed database was systematically searched for all relevant studies, regardless of language, publication date, or status, covering the period from inception to May 2025. Two authors (I.L. and T.L.) developed thorough search strategies, which were carefully adapted to account for variations in terminology and syntax (see Table 2). Additionally, reference lists were manually reviewed to identify further relevant studies. The authors of included studies were to be contacted for supplementary data if necessary.

### 2.3. Study Selection

The first (I.L.) and fifth (T.L.) authors independently and in duplicate screened all retrieved records. While they were not blinded to the study authors or outcomes, they applied a consistent method to evaluate eligibility. Any disagreement was resolved through discussion with the supervisor (A.I.T.).

### 2.4. Data Collection

Data extraction was performed by authors I.L. and T.L. using a customized data collection form. This form was designed to capture key information from the included studies, including study characteristics, design and eligibility criteria, participant details, methodological approaches, the intervention applied, and reported outcomes.

### 2.5. Risk of Bias in Individual Studies

The assessment was conducted in duplicate by I.L. and I.A.T. using the Joanna Briggs Institute critical appraisal checklist [92]. Any disagreements were resolved through discussion with A.I.T.

### 2.6. Summary Measures and Shaping of Results

As originally planned, quantitative data synthesis for meta-analysis was not carried out due to insufficient outcome data and methodological inconsistencies across the included studies.

## 3. Results

### 3.1. Study Selection and Study Characteristics

**Figure 1:**
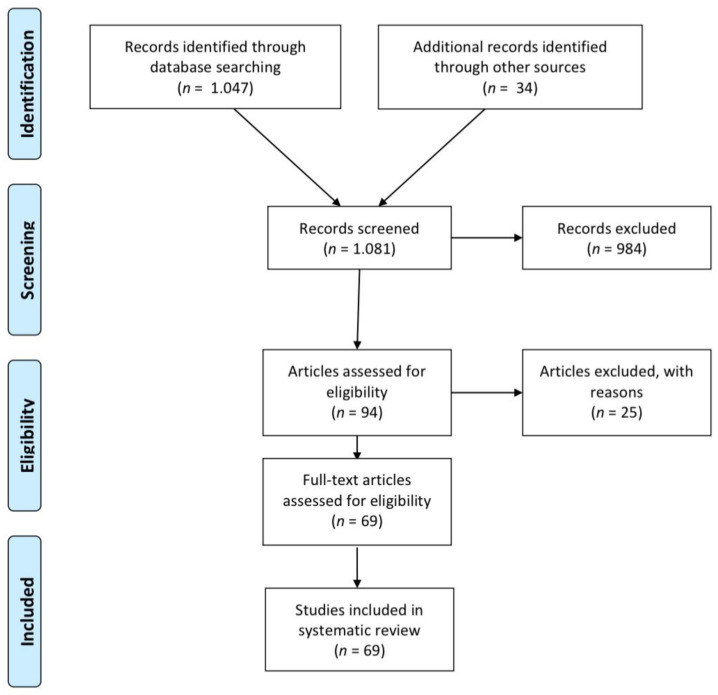
Flow of records through the reviewing process.

Figure 1 outlines the flow of the review process. The initial search yielded 1081 records, including 34 identified through reference list screening. After reading the title and the abstract, 984 records were excluded. Of the remaining 94 full-text articles assessed for eligibility, 25 were excluded, mainly due to involving orthodontic treatment. Ultimately, 69 full-text studies were included in the systematic review [18,19,32,38,57,93,94,95,96,97,98,99,100,101,102,103,104,105,106,107,108,109,110,111,112,113,114,115,116,117,118,119,120,121,122,123,124,125,126,127,128,129,130,131,132,133,134,135,136,137,138,139,140,141,142,143,144,145,146,147,148,149,150,151,152,153,154]. The characteristics of the included studies are summarized in Table 3.

### 3.2. Risk of Bias Within Studies

The outcomes of the risk of bias assessment according to the Joanna Briggs Institute’s critical appraisal checklist are summarized in Table 4. Four studies were deemed as high risk of bias [124,130,131,134], and the remaining sixty-five as being low risk of bias.

### 3.3. Results of Individual Studies

#### 3.3.1. Lateral Cephalometric Radiography

**Figure 2:**
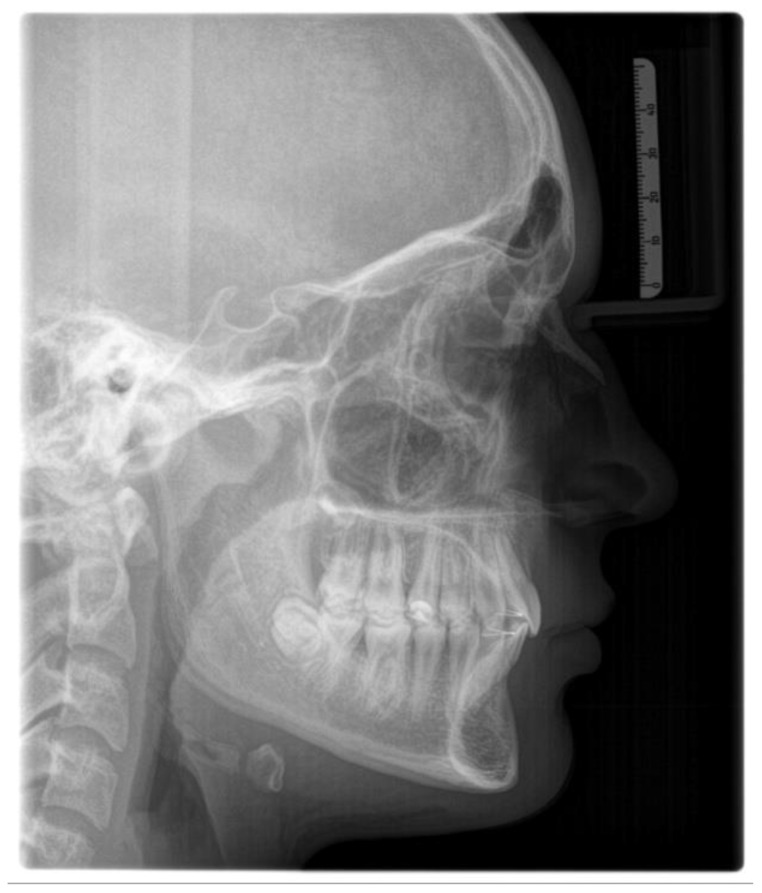
Lateral cephalometric radiograph.

The lateral cephalometric radiography (Figure 2) is virtually a static depiction of an instance throughout ongoing development [155]. It was conceptualized, described, and improved by Broadbent, who had trained under E. H. Angle served as a means to study prospectively study the changing normal human cranial anatomy [156].

**Figure 3:**
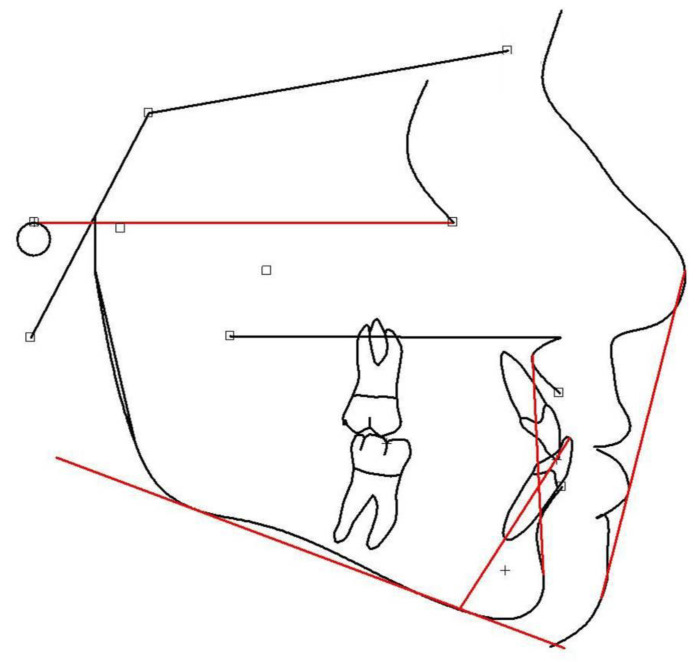
Lateral cephalometric tracing, using the Viewbox Software(Viewbox^©^ version 4.1.0.10, dHAL Software, Kifissia, Greece).

Cephalometric analysis (Figure 3) is a useful tool for predicting future facial shape [157,158,159] and undesirable deviations [160]. At large, growth prediction consists of three steps, namely: the prediction of future facial shape, being connected to the jaw relationship (the skeletal class); the estimation of the rate of growth; and, lastly, the degree of skeletal maturation in relation to the child’s age (mainly skeletal and also chronological), which may disclose whether any further orofacial change could be expected [136,161]. Potential deviations from cephalometric mean values may be evidenced following proper analysis [101,162,163,164]. Mean values have been established on boys and girls exhibiting harmonious facial types [165,166,167,168,169]. Cephalometric analysis involves drawings and calculations using specific reference points that correspond to anatomical facial and cranial structures [170].

The main reference points and planes are presented below:

Reference points:

1. Sella (S)—the center of the hypophyseal fossa (sella turcica).
2. Nasion (N)—the junction of the nasal and frontal bones at the most posterior point on the curvature of the bridge of the nose.
3. A-point (A)—an arbitrary measurement point on the innermost curvature from the maxillary anterior nasal spine to the crest of the maxillary alveolar process. A-point is the most anterior point of the maxillary apical base.
4. B-point (B)—an arbitrary measurement point on the anterior bony curvature of the mandible. B point is the innermost curvature from the chin to the alveolar junction.
5. Pogonion (Pg)—the most anterior point on the contour of the chin.
6. Menton (Me)—the lowest point on the symphysis of the mandible.
7. Gnathion (Gn)—the most outward and everted point on the profile curvature of the symphysis of the mandible, located midway between Pogonion and menton.
8. Orbitale (Or)—a point midway between the lowest point on the inferior margin of the two orbits.
9. Gonion (Go)—a point midway between the points representing the middle of the curvature at the left and right angles of the mandible.
10. Porion (Po)—the midpoint of the upper contour of the external auditory canal (Anatomic Porion) or a point midway between the top of the image of the left and right ear-rods of the cephalostat (Machine Porion).

Reference planes:

1. Sella-Nasion (S-N)—a line connecting S to N;
2. Frankfurt horizontal (FH)—a line connecting Po to Or;
3. Mandibular plane (MP)—a line connecting Go to Me;
4. *Y*-axis (Y)—a line connecting S to Gn;
5. Upper anterior facial height (UAFH)—a line connecting N to ANS;
6. Lower anterior facial height (LAFH)—a line connecting ANS to Me;
7. Nasion-A point (N-A)—a line connecting N to A;
8. Nasion-B point (N-B)—a line connecting N to B.

Each cephalometric analysis features special measurements regarding specific reference planes and/or angles, properly combining to provide the most reliable outcomes. Prominent measurements are the facial angle (it appears increased when the chin is located further forward than normal, as related to the facial profile), and the angles SNA, SNB, and ANB. The SNA angle refers to the maxillary and the SNB to the mandibular position in relation to the anterior skull base on the anteroposterior plane (SN plane). When increased, the corresponding jaw is located more anteriorly than normal. The ANB angle highlights the anteroposterior intermaxillary relationship and indicates the skeletal facial type (I, II, or III). The Lande angle relates the maxillary anteroposterior position to the facial profile. Not affected by the anterior skull base orientation, it is implemented to confirm the SNA value that could have been erroneously calculated due to the positioning of the sella (S). Actually, when the sella appears in a higher position, the SNA angle might end up increased. Moreover, an increase in the MP angle indicates a vertical facial growth pattern, while in case of a decrease, the face may appear to be growing more horizontally.

The *Y*-axis is an estimate of mandibular growth direction in reference to the Frankfurt plane. A larger angle indicates a more downward and vertical mandibular growth direction. A smaller angle indicates a mandible growing more horizontally and anteriorly. It also shows the degree of downward and forward position of the chin in relation to the upper face. Its value is commonly inversely related to the facial angle.

The ratio of the UAFH to the LAFH is more important than the individual linear measurements of UAFH and LAFH. UAFH varies with the superior–inferior dimension of the adult skull, while the ratio UAFH/LAFH indicates the balance between the facial proportions. In harmonious facial patterns, the anterior proportions range between 44 and 45% regarding the upper frontal height and 55–56% regarding the lower. The above measurements help in assessing patients who appear with open- and deep-bite. Apparently, changes in the lower anterior facial height seem to affect the optimal timing to start treatment, its duration, and the occlusal prediction throughout the mixed dentition up to adulthood [113].

Several more measurements and points of reference are available that appear interesting for predicting development, like Ricketts’ analysis (based on chronological age), which attributes a “certain” increase value to each measurement based on calculations of each individual; Johnston’s (also based on the chronological age),which uses a set of growth predictions for each cephalometric reference point; and Fishman’s (based on skeletal rather than chronological age), which directly correlates cephalometric evaluation with righthand radiography. According to Franchi (2000), Fishman’s technique for growth prediction appears more reliable [32].

Johnston (1968) compared a series of cephalograms taken overtime and concluded that the craniofacial relationships (proportions and certain angles) have greater predictive significance in comparison to the dimensions of discrete anatomical structures [93]. In addition, the same author, Johnston (1975), described a simplified method of creating long-term forecasts by the use of a printed “forecast grid” and evaluated its accuracy in a series of thirty-two 5-year forecasts [94]. Interestingly, in 1975, Schulhof & Bagha (1975) evaluated methods for growth forecasting being available at that time (the Johnston forecast grid, use of average increments of the SN line with the S as the starting point; the Ricketts’ short-range prediction method and computer forecast),and found that computer-assisted prediction was the most advantageous [96].

According to Skieller et al. (1984), four variables in combination gave the best prognostic estimate (88%) of mandibular growth rotation, namely the mandibular inclination, the intermolar angle, the lower mandibular border shape, and the inclination of the symphysis [99]. Nanda (1988) observed that open- and deep-bite subjects grow differently [101]. Deep-bite subjects are characterized by increased UAFH, whereas increased LAFH is anticipated in open-bite individuals. In addition, it was suggested that the growing pattern for each facial type is established at a very early age, before the timing of the adolescent growth spurt, even before the first permanent molar emergence. However, he did not report significant differences in the posterior face height and the ramal height between open- and deep-bite cases. Indeed, Chvatal et al., (2005) concluded that patients with extreme vertical growth might be expected to worsen over time compared with other patients [110]. Also, they claimed that horizontal mandibular remodeling does not display adolescent spurt, while the vertical growth seems to follow a sigmoidal pattern.

In 1998, Leslie et al. assessed, in a general population untreated sample, the clinician’s ability to predict with accuracy future mandibular growth using the method proposed by Skieller et al. (1984) [99,105]. Their results indicated that the information acquired from a pre-treatment lateral cephalogram was inadequate for clinically useful predictions on the degree or direction of future mandibular growth rotation. Regarding condylar growth, Buschang et al., (1999) highlighted an existing sexual dimorphism [106]. They explained that females follow a more intense rate of growth compared with males, especially during adolescence, and they reach peak adolescent velocity approximately 2 years earlier. Furthermore, they found that some individuals had little or negative growth, while others presented with more than 5 mm of yearly growth, which might explain the above mentioned individualized variability.

The Ricketts’ long-range growth prediction, applied to Turkish children by Kokadereli & Telli (1999), showed statistically significantly higher prediction reliability regarding measurements in the female facial outline, inclination and depth, their lower face height, the Condylion-point A line, their upper lip length, the angle between their palatal plane and FH plane, the angle between their mandibular plane and FH plane, their ramus height, and the so called mandibular arc angle. In males, reliable predictions could be achieved on the lower face height, the nasolabial angle, the Condylion–point A line, the facial axis, the ANB angle, and also the above-mentioned mandibular arc angle [57]. In 1999, Lux et al. showed that the predictive potential of vertical mandibular changes derived from a single lateral cephalogram seemed rather limited [107]. In their study, the angle between SN and GoMe and the facial height ratio (S-Go in relation to N-Me) were judged as inappropriate for predicting future vertical mandibular changes. In males, the measurements describing the vertical mandibular position in relation to the SN were only slightly connected with the direction of future mandibular rotation.

Turchetta et al. (2007) demonstrated that the Fishman analysis was superior to both the chronologically based Johnston grid and the Ricketts’ analysis for short- and long-term predictions because of its maturational orientation [113]. In the study conducted by Davidovitch et al. in 2016, to evaluate the Björk’s signs in extreme skeletal patterns, it was suggested that in order to classify a subject as a hypodivergent type, the clinician should assess the following in descending order: the mandibular canal curvature, the condylar inclination, the LAFH, the depth of the antegonial notch, and the interincisal angle [120]. When suspecting a hyperdivergent type, the following should be evaluated in descending order: the LAFH, the antegonial notch depth, the interincisal angle, the mandibular canal curvature, and lastly, the inclination of the condyle.

Jiwa (2020) concluded that his tested DL algorithm should not be considered equivalent with the Ricketts’ growth prediction method for a 2-year period [127]. However, he suggested that increasing the data input and training might improve the accuracy of digital prediction. Moon et al., (2022) used a multivariate partial least squares algorithm for growth prediction and they found that it seemed suitable to incorporate large numbers of variables to predict numerous landmarks in individuals [136]. After studying several statistical methods such as multiple regression analysis, least absolute shrinkage and selection operator (LASSO), radial basis function network, multilayer perceptron, and gradient-boosted decision tree to predict longitudinal craniofacial growth in a Japanese population sample, Kim et al. (2023) found that LASSO had the highest prediction accuracy for all linear and angular skeletal parameters, with values for 13-year-olds being 97.87% and 94.45%, respectively [139]. The authors claimed LASSO to be the most effective tool, overcoming the problems of feature selection and overfitting when constructing a model aimedat predicting craniofacial growth in individuals. In addition, the model had the smallest average error for all values of skeletal landmarks, linear, and angular measurements.

Parrish et al. (2023) tested seven ML models in their ability to predict post-pubertal mandibular length [141]. They found that the algorithms could predict post-pubertal mandibular length within 3 mm and the *Y*-axis within 1°. All ML algorithms yielded consistent results, with the exception of multilayer perceptron regressor, which consistently underestimated the mandibular length. For mandibular length prediction, most relevant predictive factors were the previously measured mandibular length, the chronological age, the sagittal maxillary and mandibular skeletal base position, the angle of the MP, and the face heights (anterior and posterior). The most predictive factors for *Y*-axis were the *Y*-axis value at previous timepoints, the MP angle, and the respective sagittal positions of the maxillary and mandibular skeletal bases. Wood et al. (2023) tested various ML techniques (regression algorithms) such as linear least squares, ridge, LASSO, elastic net, XGBoost, random forest, and a NN [143]. They found that all ML algorithms accurately predicted the post-pubertal mandibular length and the *Y*-axis of growth. The maxillary and mandibular lengths, and the lower face heights at earlier timepoints had the best performance as predictors for the post-pubertal mandibular length. The best predictors for post-pubertal *Y*-axis of growth were the *Y*-axis of growth, the lower face height, and the MP angle at earlier timepoints. There was not reported any statistically significant difference among the tested ML techniques, with the exception of least squares, which produced a significantly larger error in the prediction of the *Y*-axis of growth. In addition, the accuracy of the least squares model greatly increased after reducing the number of input variables. Zakhar et al. (2023) tested ML algorithms that successfully predicted the post-pubertal mandibular length within a range of 2.5 mm and the *Y*-axis within 1° [144]. Key predictors were the initial mandibular length, the chronological age, the UAFH and the LAFH, and the upper and lower incisor positioning and inclination. Important predictive factors for the *Y*-axis included previous *Y*-axis measurements, and the SN-MP, SN-Pog, SNB, and SNA angles. No statistically significant differences were noted between any of the methods regarding the 2- and 4-year forecast in mandibular length.However, regarding the *Y*-axis, the 2-year prediction resulted in significantly larger absolute deviations between the predicted and actual values compared to the 4-year prediction when using linear regression.

Zhang et al. (2023) constructed a deep CNN model, and they tested its predictive efficiency [145]. The DL model functioned well and resulted in a much higher accuracy in predicting the mandibular growth pattern in children featuring anterior crossbite. The AI model made the prediction mainly by identifying the properties of the chin, the inferior mandibular margin, the anterior tooth region, the airway and the condyle as they appear on cephalometric X-rays. Also, Larkin et al. (2024) suggested that short-term growth prediction might be possible in pre-adolescent patients with Class I malocclusion using a CNN algorithm and longitudinal lateral cephalograms for a 2-year growth prediction [148]. However, they stated that further studies are needed to improve the prediction accuracy using hard-tissue and soft-tissue landmarks of the chin area. Moon et al. (2024) found that the multivariate partial least square (PLS) regression method and a DL algorithm (TabNet-based deep NN) seemed to be valuable tools for predicting craniofacial growth [151]. At large, the TabNet AI algorithm predicted growth more accurately than the PLS method, particularly for those landmarks in the maxilla and mandible (on average, AI showed lower error by 2.11 mm compared with the PLS). However, the PLS method behaved better in predicting landmarks with low variability. Among 78 landmarks, AI was more accurate in 63, whereas PLS was more accurate in 9. The remaining six landmarks showed no statistical difference between methods. Overall, soft-tissue landmarks, mandibular landmarks and vertical growth involved, respectively, greater prediction errors than hard-tissue landmarks, maxillary landmarks, and growth changes in the horizontal direction. Therefore, applying AI for growth prediction might be more advantageous when uncertainty is considerable. Myers et al. (2025) trained three ML models (LASSO regression, random forest, and Support Vector Regression) to predict long-term growth-related changes in skeletal and dental relationships [153]. They reported on the AI models’ ability to predict post-pubertal maxillary values, upper and lower incisor positions, and upper face height, with a clinically acceptable margin of 2 mm or 2°. Prediction accuracy was higher for skeletal compared to dental relationships. The pre-pubertal measurement values and the sex proved consistently the most significant predictors of post-pubertal growth.

#### 3.3.2. Cervical Vertebrae Radiography

**Figure 4:**
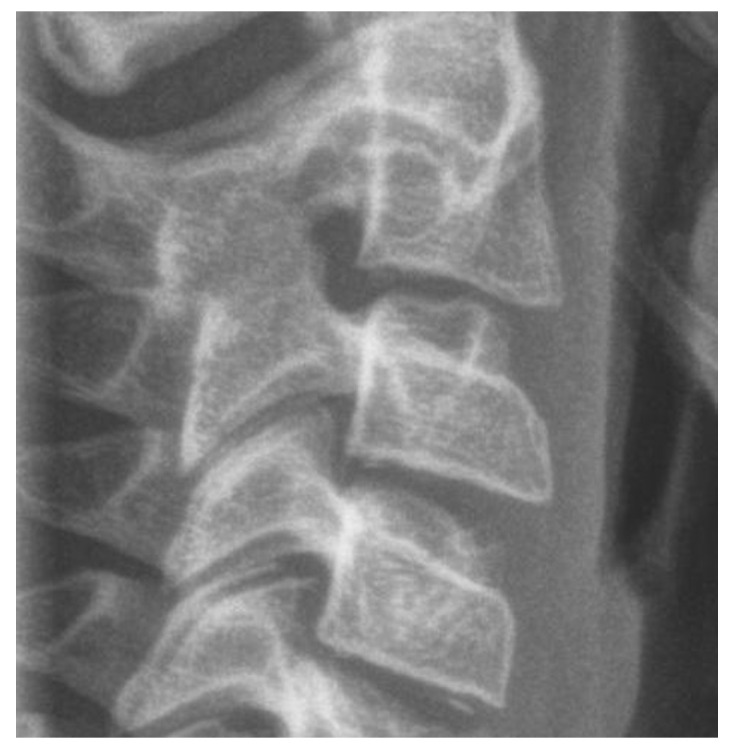
Cervical vertebrae radiograph—rectangular horizontal shape.

**Figure 5:**
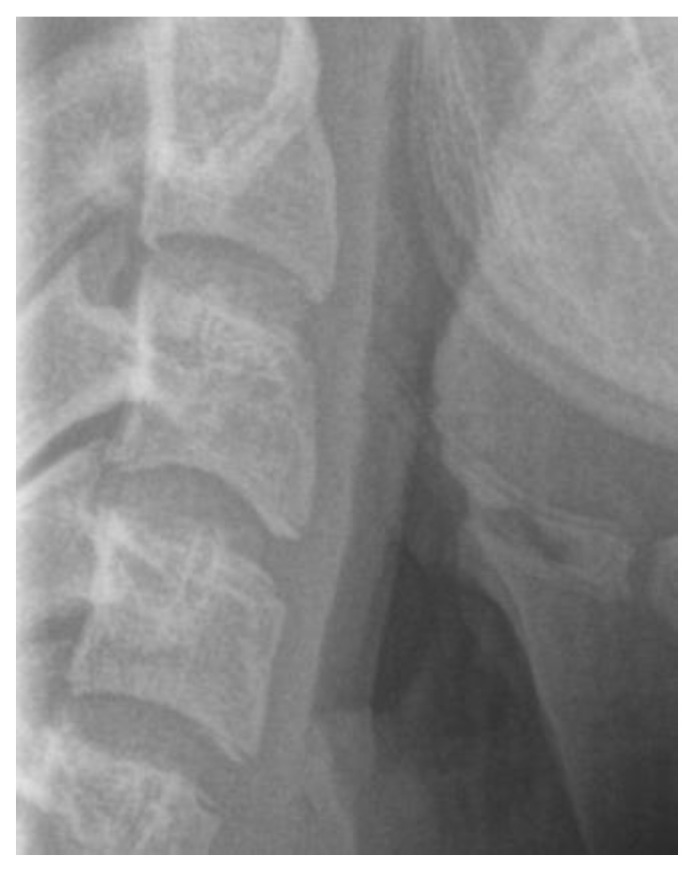
Cervical vertebrae radiograph—square shape.

It is a reliable radiographic technique that evaluates facial skeletal maturity through the developmental stage of the cervical vertebrae (Figure 4 and Figure 5) [32,129,152]. It was originally conceived and developed by Lamparski in 1972 [171]. According to Bulut & Hezenci (2024) and Caldas et al. (2007), cervical vertebral maturation (CVM) appears appropriate for the appraisal of skeletal maturity in individuals and the estimation of their skeletal age on the basis of a single cephalometric observation, without additional X-ray exposure [142,146,172].The accuracy of the method in detecting the timing of the pubertal growth spurt may prove useful for treatment planning in cases of developmental orthodontic deficiencies [32].

**Figure 6:**
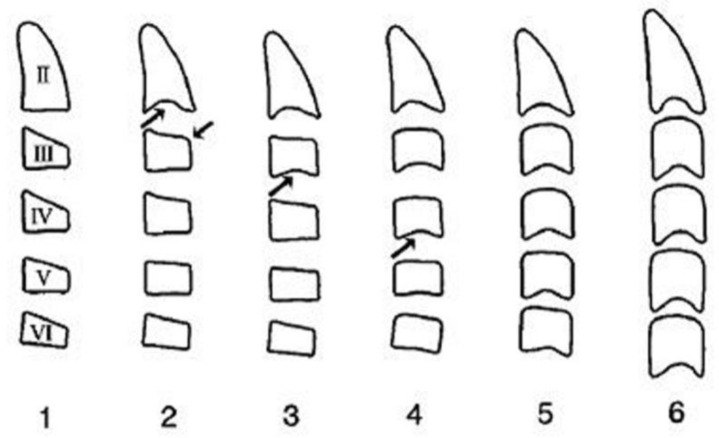
Cervical vertebrae radiograph—stages.

The cervical vertebral lower surfaces are assessed for changes as they transition from flat to concave, and the ratio between their height and width is also calculated (Figure 6). Six consecutive stages of CVM become evident in cephalometric radiographs, regardless of chronological age and gender [171]. Initially, (stage 1) the inferior surface of the cervical vertebrae is flat while the superior border is tapered from posterior to anterior. Further patient mandibular growth might be anticipated and, according to Baccetti et al. (2002), the peak in mandibular growth will occur in more than one year after this stage [109]. Next, a depression forms at the inferior border of the second vertebra, its body anterior vertical height increasing (stage 2). Allegedly, at this particular age, the remaining time up to the growth peak is approximately one year [125]. At the third stage, a concavity develops at the inferior border of the third vertebra. On average, the peak of mandibular growth is imminent [125,128]. Actually, the peak in skeletal growth occurs between the CVM stages 3 and 4 [32,173]. Although statural height greatly increases between CVM stages 1 and 2, and up to stages 3 and 4, it is noteworthy that the increase in mandibular length (as depicted by the distance Co-Gn) is more pronounced between CVM stages 3 and 4 [32]. In CVM stage 4, the lower surface of the fourth cervical vertebra develops a depression, and further depressions begin to form at the fifth and sixth stage. All vertebral body shapes appear rectangular in shape. The patient is less than 1–2 years past the maximum mandibular growth [108]. In CVM stage 5, concavities have become evident at the lower body borders of all the cervical vertebrae. The bodies are nearly square in shape and the intervertebral spaces measure diminished. The individual is one year past the age of maximum skeletal growth and thus, more mandibular vertical than anteroposterior growth is to be expected [32]. Lastly, at CVM stage 6, all vertebral lower surface cavities have deepened, and their shape is greater in height than in width. The person is 2 years past the mandibular growth peak. Allegedly, the aforementioned chronological consecutive vertebral shape changes must be interpreted with caution due to underlying gender dimorphism [125,129,146]. In particular, Madiraju & Almugla (2024) suggested that the cervical vertebral method of predicting the mandibular growth potential was applicable only to young Saudi males, and that the chronological age correlated statistically significantly with skeletal age only in these young males [149].

The above-mentioned method to detect the peak in mandibular growth was modified by Baccetti et al. (2002) [108], who suggested assessing only the second through the fourth cervical vertebrae as they appear on a single cephalogram. The new method introduces five maturational stages instead of six, the peak in mandibular growth occurring between CVM stages 2 and 3. Stage 5 happens at least two years after the peak of growth. In the new version, skeletal maturity can also be appraised on a single cephalogram and through the analysis of only three vertebrae, usually well discernible despite the use of a protective radiation apron with collar [108]. Furthermore, Yamaguchi et al. (2024) developed a technique to predict mandibular size that is based on the changing morphology of just the fourth cervical vertebra [152].

In addition, a quantitative computer-based method has been tested to track the body’s anatomical changes in just four maturing cervical vertebrae. The model also incorporated details on the sex and the age, yielding satisfactory and reproducible measurements on skeletal age prediction [118]. In addition, Cericato et al. (2016) combined observations on CVM with details on the developmental stages of mandibular teeth (excluding third molars) to estimate skeletal age in 7–16-year-old youngsters. They argued on the reliability of the technique but also called for further research [119]. Franchi et al. (2021) concluded that the development of the cervical vertebrae could be used to reliably predict the pubertal peak in mandibular growth [128]. On the contrary, Hosseini et al. (2016) claimed that the clinical appraisal of CVM may not correlate consistently with the level of mandibular growth due to the variability in its pattern. Eventually, they alleged that skeletal maturity may prove more useful in orthodontic research involving a sample, as it lacks adequate predicting power for individuals. Also, the technique may not fully agree with other radiographic methods determining skeletal age and this can be attributed to the impact of environmental variables, the ethnicity, and the gender [174]. Jeon et al. (2021) suggested that evaluating skeletal maturity in six stages is of limited accuracy and thus called for alternatively determining the maturity of the radius, the ulna, and the carpal short bones in cases requiring estimation of the bone age or any amount of residual growth. They recommended additional radiographs (such as a hand–wrist X-ray) for more detailed evaluation, as needed [129].

Similarly, Flores-Mir et al. (2006) studied the three cervical vertebrae (second, third, and fourth) as they appear in the cephalometric radiograph and reported on its validity and the advantage in relation to reduced radiation exposure [111]. Also, Uysal et al. (2006) concluded that it is appropriate to use the cervical vertebral skeletal maturation method in daily orthodontic diagnostic practice, at least when practicing with a sample of Turkish origin. In their study, chronological age and CVM were found to be highly correlated regardless of sex, although females performed statistically significantly better [37].

Cephalometric radiographs have been used to draw reference points and lines involving the cervical vertebrae [103,104]. Also, the inclination of the cervical column has been used for predicting the positioning of the temporomandibular joint (TMJ), the dimensions of the jaws [103], and the facial height [104]. In particular, the position and the inclination of the upper spine were correlated with the craniofacial complex, and its subsequent increase. A statistically significant relationship was recognized between the cephalo-cervical inclination and the final facial shape, particularly regarding the lower anterior height. Children at the age of nine years exhibiting a slightly more posterior skull position and a narrower angle between the upper spine and the skull may end up with more limited posterior movement of the temporomandibular joint (TMJ), increased palatal length, and a tendency for increased horizontal mandibular growth. Moreover, in adolescence, they may appear with bimaxillary prognathism. An opposite pattern may occur in case of upward head position and a greater craniocervical angulation. These adolescents show pronounced posterior displacement of the TMJ, reduced palatal length, and anterior mandibular displacement. In sum, a smaller angle between the skull and the upper vertebrae is associated with more forward craniofacial increase, while a larger angle is connected with more vertical increase [103,104,175].

AI has been increasingly implemented to determine the skeletal age [130] and the pubertal growth spurt [87] with the aid of the cervical vertebrae. Kök et al. (2019) tested seven different ML algorithms to determine the cervical vertebral developmental stages. It was found that they exhibited varying levels of prediction accuracy, while the ANN proved the most stable [124]. Amasya et al. (2020) developed and compared five ML algorithms to analyze cervical vertebrae and showed that the ANN model performed better in classification than the rest (decision tree, random forest, logistic regression, and support vector machine) [176]. The above ANN model was compared with four independent human observers regarding the recognition of the respective CVM stages; but on average, there was just 58.3% of agreement [176]. Zhou et al. (2021) reported on the development of a CNN model to assess the CVM. AI showed a good agreement with human examiners. It was found that the mean measurement error between AI and humans was 0.36 *±* 0.09 mm, whereas the mean error between human examiners was 0.48 *±* 0.12 mm. The agreement between AI and the gold standard was good (the correlation coefficient was up to 98%). Also, the accuracy in CVM staging was 71% [132]. Seo et al. (2021) compared six unsupervised DL-based CNN models and implemented a gradient-weighted class activation map (Grad-CAM) to visualize them. Their findings indicated that all the algorithms achieved an accurate rate of over 90%, and Inception-ResNet-v2 had the best performance at accuracy of 0.941 *±* 0.018%. In addition, the above algorithm, in particular, focused on several cervical vertebrae, whereas most of the others had mainly focused on the third cervical vertebra. Hence, it was concluded that the use of DL models in clinical practice could aid dental practitioners in making accurate diagnoses and treatment plans [142]. Li et al. (2022) concluded that CNN models are a convenient, fast, and reliable method for CVM evaluation. They tested four CNN models, namely, VGG16, GoogLeNet, DenseNet161, and ResNet152 to find that the ResNet152 proved as the most suitable for maturation analysis (accuracy of 67.06%) [135]. Attici et al. (2022) applied unsupervised DL to identify and classify the stages of CVM and proposed a custom-designed CNN model with a built-in set of directional filters that highlight the radiographic edges of the cervical vertebrae. Their model had a validation accuracy of 84.63% (removing the filters, the custom-designed CNN model’s test accuracy decreased to 80.75%) in maturation stage classification, more than the accuracy of other investigated pre-trained DL applications (MobileNetV2, ResNet101, and Xception) that were also used with directional filters, attaining accuracy of 74.10–80.86%. They concluded that the DL model of interest can be used for determining the skeletal maturity stage and the timing of treatment, especially by clinicians with less experience [134]. Similarly, Mohammad-Rahimi et al. (2022) introduced and evaluated a novel DL model to evaluate cervical vertebral maturation [177]. The model’s validity for the six-class CVM was 62.63%, and for the three-class, it was 75.76%. The test diagnostic accuracy was 61.62% and 82.83% for the six- and three-class, respectively. In addition, substantial agreement was observed between the evaluating orthodontists and the AI model. In conclusion, the newly developed AI model was reasonably accurate in detecting the skeletal age and had a high reliability in detecting the pubertal stage, but its accuracy measured lower compared to human reviewers. Radwan et al. (2023) investigated lateral radiographs using applications based on CNNs and deep NNs to assess the developmental stages of the cerebral vertebrae. They concluded that their algorithm performed well at the pre-pubertal (F1 score 76%) and post-pubertal (F1 score 90%) stages, but scored an F1 value (an important measurement that sums up the predictive performance of any model in machine learning by combining two otherwise competing metrics such as precision and recall) of only 57% at the pubertal stages. This means that the algorithm classified pubertal stage erroneously in nearly 40% of cases. However, the inter-observer measurement correlation showed almost perfect agreement, indicating high reliability and reproducibility. Hence, the authors suggested that AI can be used to determine skeletal age in the clinical setting [137]. Li et al. (2023) tested a fully automated, DL-based, high-accuracy CVM assessment system called the psc-CVM assessment system to acquire information on the period of growth. Overall, the system’s total accuracy was 70.42%. The weighted agreement between the system and the expert panel was 0.844, and so the program was judged as highly accurate. The digital system proved significantly consistent with expert panels in CVM assessment, an indication that it can be used efficiently and accurately as clinical aid for determining skeletal age [140]. Seo et al. (2023) devised a DL approach to estimate bone age by studying the cervical vertebrae on lateral cephalograms of growing children using DeepLabv3þ and InceptionResNet-v2 architecture. The model featured average accuracy and mean F1 scores of 0.956 and 0.895, respectively, for the partition of cervical vertebrae. Seemingly, the proposed method can estimate bone age with sufficient accuracy based on lateral cephalography [142]. Khazaei et al. (2023) found that a CNN based on the ConvNeXtBase-296 architecture exhibited high accuracy in assessing the pubertal growth spurt based on CVM staging in both three-class (82% accuracy) and two-class (93% accuracy) methods of assessment [138]. Mohammed et al. (2024) suggested that the level of CVM can be used accurately and reliably for the estimation of skeletal age in growing children. To test the method, they selected an interdisciplinary CNN-based DL approach. In males, the model demonstrated a high degree of accuracy (98%), and so each developmental stage can be predicted. Also, the model may improve by integrating the chronological age as derived from the level of second permanent molar calcification in an orthopantomogram [150]. Nevertheless, Yilmaz & Gonca (2025) suggested that vertebral skeletal age as estimated by ML-assisted statistical testing in Turkish children and adolescents did not demonstrate any clinical advantage over chronological age. Moreover, vertebral skeletal age estimation showed greater variability in boys than in girls. These authors admitted that the methods in their study provided limited useful information on the timing of orthopedic treatment or in defining the end of growth. They advised clinicians to carefully consider average gender differences regarding chronological and skeletal ages when needing to calculate the correct timing for starting treatment [154].

#### 3.3.3. Hand–Wrist Radiograph

**Figure 7:**
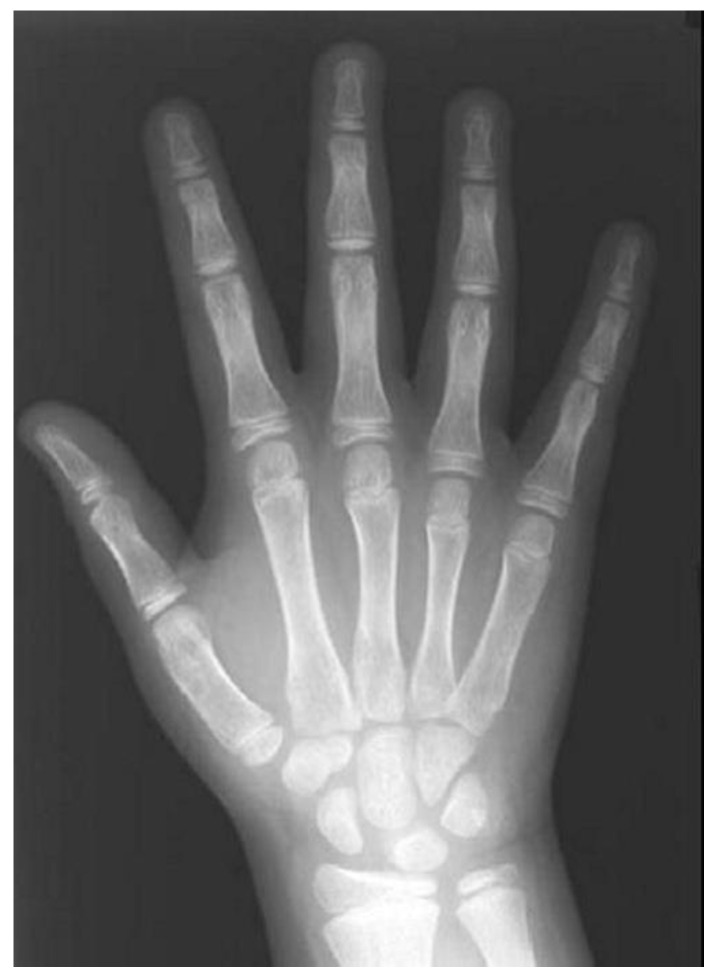
Hand–wrist radiograph.

The hand and wrist radiograph (HWR) has been used to reliably evaluate the skeletal age of growing children and adolescents, (Figure 7) and to determine their status of physical maturity [19,178,179]. Every person matures according to a particular schedule that can be disclosed by the HWR [19]. However, some authors have expressed reservations on the relationship between skeletal assessment by HWR and maxillofacial development [20]. In addition, conclusions on skeletal maturity should not be drawn solely from isolated ossification events because the procedure might be affected by hormones and sexual dimorphism [180].

A HWR exposes the child to low radiation, less than 0.00012 mSv [181], resulting in a minimal relative risk of 40-year mortality of 5.1 × 10^−8^ [182]. Three standardized methods have been developed and become more popular worldwide in the assessment of skeletal age using the HWR, namely, the Greulich–Pyle method, the Tanner–Whitehouse, and the Fels method.

Greulich and Pyle evaluated the HWRs obtained from about 1000 white male and female individuals of medium to high social classes at Cleveland (Ohio, USA), which illustrated different phases of bone maturation between 0 and 19 years of age [40]. Their atlas features a series of HWRs in consecutive stages of ossification belonging to ordinary, healthy children and serves to compare and indicate the particular skeletal age of individuals [183]. The Tanner–Whitehouse (TW) method was initially developed from a sample of European children. Progressively, this system has been modified and revised moving from the initial edition known as Tanner–Whitehouse method 1 (TW1) to the revised methods known as TW2 and TW3. Overall, the Tanner–Whitehouse method is accurate, but more complex and time consuming [184]. The Fels method was developed by Roche through a longitudinal study, based on a total of 13,823 serial X-rays of the left hand and wrist belonging to 355 male and 322 female children born between 1928 and 1974, aged of one month up to 22 years [185]. Although this method is very accurate and allows for young children’s bone age estimation, it is quite complex for daily use.

The section of the skeleton appearing in the HWR is composed of 27 small bones (carpals, metacarpals, and digit phalanges) and the distal ends of two long bones: the radius and the ulna. The development of the upper distal limb occurs throughout the entire postnatal growth period to provide a useful way of assessing osseous maturity [19,186,187].

Research has demonstrated that there is not any statistically significant difference in the degree of development between hands. Therefore, the radiographic study of either upper limb is equally reliable [188]. In addition, in cases of dissimilar maturation between hands, an average value could be calculated and used.

Important nuclei of ossification are considered the sesamoid, the epiphysis of the middle phalanx of the middle finger, and the epiphyses of the distal phalanges. The sesamoid is traced at the inner/upper part of the thumb metacarpophalangeal joint, on average 0.3 years in boys (standard deviation 0.1 years) and 0.5 years in girls (SD of 0.2 years) before the pubertal growth peak. The middle phalanx epiphysis of the middle finger has the same width as the corresponding diaphysis 2.4 years before the growth peak in boys (SD of 0.3 years) and 2 years in girls (SD of 0.3 years). It is shaped like an inverted cup and almost covers the corresponding diaphysis in width, 0.3 years following the growth peak in boys (SD of 0.1 years) and 0.6 years in girls (SD of 0.2 years). Synostos is occurs 2.3 years after the growth peak in boys (SD of 0.2 years) and 2.8 years in girls (SD of 0.2 years). The epiphyses of the distal phalanges ossify 1.6 years after the peak of growth in males (SD of 0.1 years) and 1.5 years (SD of 0.2 years) in girls [97,189,190].

The onset of ossification of the sesamoid and the pisiform bones, and the appearance of the hook of hamate have been advocated as reliable indicators of the onset of puberty [38,97,191,192].

Leite et al. (1987) found that skeletal age assessment using the ossification events of the hand’s first, second, and third fingers, is adequately valid for clinical purposes as when using the entire hand–wrist. This area of the hand was selected because it can be included in the lateral cephalometric radiography, thus reducing the need for additional exposure [100]. To determine maturity, the above authors focused on the sesamoid and the epiphyseal-diaphyseal stages of ossification. Comparing similar methodologies, when using only the three fingers, skeletal age assessment will be slightly advanced (mean difference of 2.1 months) regardless of sex and the timing of observation. On average, in boys, the three-fingers method does not deviate from that of the ordinary hand–wrist by more than 2.89 months (minimum deviation of 0.32 months); whereas for females, the greatest deviation increases to 4.45 months (minimum of 1.55 months). The differences emerge because throughout normal maturation, ossification proceeds from the first to fifth fingers and from distal to proximal phalanges. The maximum deviations occurred during the time of epiphyseal-diaphyseal fusion, when growth is ending, and thus are considered as, clinically, less important [100].

The peak of growth occurs at about the time of epiphyseal capping of the fingers and radius, and the ossification of the sesamoid and hamate. If the sesamoid is not ossified, the peak rate of growth has not yet been reached. Most children enter the pubertal spurt when the sesamoid becomes visible. Nevertheless, occasionally its ossification may start just after the peak. Growth deceleration is indicated by epiphyseal union at the third finger, progressing from distal to the proximal phalanges, and at the radius [97,189].

According to Taranger & Hägg (1980), the peak of growth occurs at about 10 years of age in females and 12.1 years in males and ceases at 14.8 and 17.1 years, respectively. In both sexes, the peak increase in height occurred two years after the onset of the pubertal spurt. Although the pubertal growth spurt occurs earlier in girls than in boys, there is no difference in its duration. On average, girls stopped growing at 17.5 years of age and boys stopped at 19.2 years [19,38,193].

Mohammed et al. (2014) reported statistically significant (*p* < 0.05) sex differences between chronological age and the observed skeletal maturation as maturation comes earlier in females. On average, males begin to mature skeletally at 10.17 ± 1.59 years of age, whereas girls start at 9.98 ± 1.4 years. Females complete skeletal maturation at the age of 17.34 ± 1.67 years and the males at 18.1 ± 1.13 years (*p* < 0.0001) [117]. Hosseini et al. (2016) evaluated the hand–wrist bones in boys and girls and reported that females were more advanced at all age groups [174].

Moreover, according to Fishman (1982), it is not uncommon to observe the greatest rate of pubertal facial growth at a slightly later age compared to the occurrence of the maximum rate of statural increase [19]. Individuals demonstrate noteworthy variation in their maturation and early patterns may not persist in adolescence, when unexpected changes are likely to emerge. He also claimed that the rate of body growth is connected to developmental findings on HWRs. Also, it was concluded that maxillary and mandibular variations are associated with changes in the maturing skeleton, although the jaws reach their maximum growth rate later in comparison to body height, which develops more rapidly than the facial dimensions in middle and late adolescence. The maxilla ceases to grow earlier compared to the mandible, although the latter tends to catch up. It was found that females exhibit a higher proportion of growth during early adolescence, but both sexes show similar percentages of completed development after the timing of maximum growth. Early in adolescence, the speed of statural growth exceeds that of the face, with the greatest velocity for both occurring before the peak. Females grow faster and appear with earlier statural and maxillary maturation, whereas males feature higher mandibular growth velocity. Eventually, after the observed peak, the rate of growth diminishes more rapidly in females [19]. Late mandibular growth spurt, often attributed only to boys, shows a particular sex difference only when chronological measurement is used. On a maturational scale, boys do not demonstrate substantial differences from girls in the length of growth increments. Simply, they are older when these stages occur [38].

The HWR prediction method was reported to be moderately highly, statistically significantly (*p* < 0.05) correlated with findings regarding the maturing skeleton in radiographic pictures of the cervical vertebrae. The correlation values measured 0.70–0.87 depending on the stage of individual maturity [111]. Uysal et al. (2006) also found that both techniques were statistically highly correlated (correlation with the sexes combined was 0.86, correlation separately for males and females was 0.78 and 0.88, respectively, *p* < 0.001) [37]. Bulut & Hezenci (2024) reported a statistically significant correlation between the hand–wrist and CVM stages (*p* < 0.001). The correlation coefficient was 0.831, 0.831, and 0.760 in Class I, II, and III females, respectively. In males, it was 0.844, 0.889, and 0.906, respectively. When sex and malocclusion were not considered, the correlation was 0.887. The highest correlation emerged in Class III males, and the lowest in Class III females. Due to female differences according to malocclusion types at different stages of skeletal maturation, growth assessment should proceed carefully. It was asserted that the CVM method can be used for pubertal growth spurt assessment without HWR [146]. On the other hand, Hoseini et al. (2016) reported a low level of agreement between HWR and CVM evaluation (46.7% agreement, coefficient of 0.312), slightly higher in males (0.33) compared to girls (0.27). The methods had the highest agreement in 8- and 9-year-olds and the lowest in 12- and 14-year-olds. Thus, the level of agreement was only acceptable in 8- and 9-year-olds of both genders and in 10-year-old boys. Interestingly, the authors alleged that the methods should not be used alternatively [174].

In males, the skeletal age had been determined from HWRs with the Greulich–Pyle method before the pubertal growth spurt (at 9, 10, or 11 years of age) does not necessarily predict the timing of peak mandibular growth [112]. Hence, it was suggested that it is better to treat developmental discrepancies around the average age when the peak mandibular increase is anticipated.

Verma et al. (2009) found that in both sexes, there was a statistically significant correlation (*p* < 0.001) between body height and growth prediction as assessed from HWRs. The increase in mandibular body length showed the greatest correlation with growth prediction, but this association would not allow a reliable growth prediction. There was statistically insignificant correlation between the growth of the cranial base, the maxilla, and growth prediction based on HWRs. The authors commented on the special growth pattern of each individual and also highlighted that the various craniofacial structures feature different growth potentials. Therefore, they questioned the reliability of HWRs for quantitative craniofacial growth prediction. Nevertheless, when attempting to assess the timing of growth in an individual, the HWR can assist in treatment planning [194]. In addition, the same authors concluded that it is not possible to predict the vertical growth potential of the mandibular ramus at the beginning of orthodontic treatment by solely consulting HWRs and any cephalometric variable other than the initial ramus height [194,195].

Sato et al. (2001) tested the predictive potential of a computer-assissted skeletal maturation assessment tool with the aid of the TW2 method, calculating special mathematical equations with anatomical measurements. Despite the underdevelopment of digital technology at the time, they concluded that computers may be adequately reliable in assessing the skeletal age and the mandibular growth [55].Gonca et al. (2024) used ML algorithms, namely fractal dimension (FD) analysis, with HWRs to predict the skeletal maturation stage. The bone structure has fractal properties and can be evaluated using FD analysis to evaluate physiological changes and determine maturation and pubertal growth. FD analysis transforms the complex structure of an image into a FD value by simplifying it through various image processing procedures. The above authors concluded that the method was not sufficient for prediction, although the growth predicting reliability could improve significantly if it was supplemented with data concerning the sesamoid ossification, the age, and the sex [147].

The HWR has been used successfully to estimate the skeletal age of individuals in growth studies evaluating various other methods, due to its reliability and convenience of application [26,55,103,118,121,123,154,196]. In particular, Kim et al. (2021) implemented ML techniques on radiographic images of cervical vertebrae aiming to predict the CVM stages. Their ground truth data were also based on findings from HWRs [130].

#### 3.3.4. Metal Implants

The method, introduced by Björk [197], has been used in research and comprises radiographic imaging after inserting metallic implants in the jaws to be used as reference points for the precise superimposition of consecutive cephalometric radiographs [18]. Thus, cephalometric planes with changing dimensions and orientation are used for drawing conclusions on facial remodeling and the pattern of growth [59]. Mini screws have been initially tested in animal models [198]. The implants are tantalum pins 1.5 mm long and 0.5 mm in diameter and are inserted under local anesthesia using a special instrument without the need to expose the bone [199]. Three to four implants are placed in each jaw, are well tolerated by the subjects, and do not cause tissue inflammation. Sadly, the implants can be rejected or displaced within the jaw, mainly due to either erroneous technique of placement or misjudgment in the planning. There are accounts of implants ending up inside the nasal cavity due to nasal floor resorption, injuries to the periodontal ligament or the dental root, nerve involvement, subcutaneous emphysema, and cases of soft tissue ulceration or inflammation [200].

In his seminal implant study, Björk (1969) claimed that in younger children, it gets more difficult to predict the final facial shape using morphometric analysis based on a single cephalometric radiograph. Throughout the period of growth, the implant technique can detect considerably greater alterations in the vertical jaw relationship than other conventional methods, and can also locate sites of growth and resorption of individualized intensity [18]. In addition, his research has demonstrated that the human mandible grows essentially at the condyles, usually not in the direction of the ramus, but slightly forward, not always linearly in direction, usually bending slightly backwards. Overall, the anterior aspect of the chin exhibits stability, lacking extreme growth potential in healthy, ordinary subjects. By contrast, symphyseal thickening happens by bone apposition on its posterior surface. Also, on the inferior border there is osseous apposition, increasing the symphyseal height. Normally, under the mandibular angle there is resorption, which may be pronounced. However, bone apposition may happen on the angular lower border. Combined osseous apposition and resorption results in the individualized shaping of the lower mandibular border, and so the pattern of growth becomes evident. Sadly, a prediction of subsequent growth is made on the assumption that the trend will remain unchanged. The mandibular canal is not remodeled to the same extent as the outer surface of the jaw, so the trabeculae related to the canal remain relatively unchanged. The lower border of a developing mandibular molar germ appears rather stationary until the roots begin to form. Thus, this germ may serve as natural reference in mandibular growth analysis [18].

The mandible may be regarded as being more or less unconstrained, and may change its inclination in different ways, depending on the center of rotation, which may be located at the posterior or anterior of the bone or somewhere in between. The spatial remodeling about the centers of the TMJs may culminate in deep-bite and anterior face height underdevelopment. Forward mandibular rotation at a center located at the lower anterior teeth incisal edges produces marked posterior facial height development and more normally increasing anterior height. Then, the posterior part of the mandible rotates away from the maxilla. Importantly, extreme mandibular rotation affects the path of dental eruption. This should be assessed in the planning of treatment due to the increased risk for extreme migration after extractions, needing more robust anchorage [18].

Björk (1969) suggested seven structural signs of extreme mandibular remodeling in relation to condylar growth direction. Not all of them exist in a particular individual, but the greater the number, the more reliable the prediction will prove. Unfortunately, these features are not fully developed before puberty. They are connected with the condylar head inclination, the mandibular canal curvature, the lower mandibular border shape, the inclination of the symphysis, the interincisal angle, the interpremolar or intermolar angles, and the anterior lower face height [18].

Mitchell et al. (1975) recruited some children in whom gold implants had been inserted in the mandible to investigate whether the Ricketts’ theory of arcial growth could be of use for mandibular morphological prediction [95]. The principle of arcial growth of the mandible, proposed by Robert Ricketts, suggested that mandibular growth occurs along a curve that is a segment of a circle. Allegedly, this growth quotient mainly happens by vertical bone formation at the ramus, leading to an upward and forward movement of the occlusal plane and the teeth [201]. Eventually, Mitchel et al. found that the arcial growth theory appeared valid for mandibular growth prediction, but they called for supplementation with HWRs to increase the technique’s accuracy on the prediction of anticipated remaining growth.

Skieller et al. (1984) investigated the potential of predicting the direction and the extent of mandibular growth after appraising morphologic variables on a single profile radiograph taken at puberty. Their selected sample featured extreme growth patterns and had previously participated in an implant study [202]. Four variables in combination gave the best prognostic estimate (88%), namely the mandibular inclination (comprising the proportion between posterior and anterior facial height, the lower gonial angle, and the lower border inclination), the intermolar angle, the shape of the mandibular lower border, and the symphyseal inclination [99].

To evaluate the Björk’s prognostic method in extreme skeletal patterns, Davidovitch et al. (2016) studied pre-treatment orthodontic cephalometric radiographs of post-growth subjects. They suggested appraising either the curvature of the mandibular canal, the condylar inclination, the lower anterior facial height, the antegonial notch depth, and the interincisal angle or the lower anterior facial height, the antegonial notch depth, the interincisal angle, the mandibular canal curvature, and the condylar inclination in case of suspected hypodivergent or hyperdivergent type [120].

Currently, the use of metallic implants has been largely demoted by three-dimensional imaging and computed tomography [203,204]. However, implants are still applied in animals for research purposes, and their components are being investigated in vitro to confirm their safety, and to investigate their properties [205,206,207].

#### 3.3.5. Other Methods

According to Perinetti et al. (2011) and Perinetti & Contardo (2016), the gingival crevicular fluid alkaline phosphatase enzyme (GCF-ALP) can be considered adjunctly as a valid, non-invasive biomarker to disclose the timing of the pubertal growth spurt in periodontally healthy subjects. However, standard radiographic methods should also be prescribed. Apparently, the enzymic activity doubles in the timing of the pubertal growth spurt compared to pre-pubertal and post-pubertal developmental stages. Therefore, the GCF-ALP activity might have diagnostic potential for identifying the optimal timing for orthognathic or functional treatment of various maxillofacial disharmonies in growing patients. The sampling and laboratory analysis of the GCF-ALP are readily available, very simple, cheap, and quick, and can be performed in a clinical setting [115,122]. Indeed, combining information on the patient’s chronological age with salivary ALP activity may enhance the method’s predictive value [208].

Rossouw et al. (1991) demonstrated statistically that the size of the frontal sinus, as appearing on a lateral cephalometric radiography, is a useful additional indicator of continuing mandibular growth [102]. Ruf & Pancherz (1996) found a rather high degree of accuracy in predicting skeletal maturity by analyzing the frontal sinus development as depicted on lateral head films. Correct predictions were possible in 75 or 85% of the subjects depending on the interval between the radiographic examinations (1 or 2 years, respectively). Although the authors did not advocate the replacement of traditional methods for the assessment of skeletal maturity, it was proposed that appraising the sinus can provide important information during orthodontic treatment [196].

Sadly, the mandibular antegonial notch, easily measured by cephalometric radiography, has been suggested by Kolodziej et al. (2002) to be insufficient to indicate future growth consistently. It was explained that despite the statistically significant negative relationship between the depth of the notch at adolescence and the horizontal maxillary and mandibular growth trend, the correlation is clinically insignificant and thus of little value for indicating future facial growth in average populations [109].

Abate et al. (2022) suggested that in young adults with remaining vertical growth potential, a larger maxillary sinus may be related with future vertical growth. They found an inverse relationship between ANB and the size of the sinuses. A significant association was evident between depth, surface, and volume of the sinus, and the mandibular protrusion. Moreover, the size of the sinus was found to increase statistically significantly with both the total anterior vertical dimension, and also the respective upper and lower dimensions. Lastly, the dimensional increase in the sinus corresponds to a lengthening of the skull base [133].

Nawaya & Burhan (2016) found that in males and females, the coronal structure of the first and second permanent mandibular molars, as it appears on a panoramic radiography, can predict the timing of pubertal growth stages with consistency [121]. According to Issa et al. (2017), the immature apex of the permanent mandibular canine appearing on a panoramic radiography could indicate the pubertal growth period, in both sexes [123]. This is in agreement with the findings of Nayak et al. (2010) and Hegde et al. (2014) that the calcification stages alone of the mandibular permanent canine that are easily tracked on customary dental periapical films were highly statistically significantly correlated to the maturation of the skeleton. It is important that the Dermirjian’s middle stages of calcification of the aforementioned tooth indicated approximately 80–100% of remaining pubertal growth; the penultimate stage showed 25–65% of remaining growth, whereas the end of the canine’s calcification reliably indicated the cessation of the pubertal growth spurt. On the other hand, the correlation with chronological age proved statistically insignificant [114,116].

Biochemical methods involving the administration of radioactive isotopes are applied to study alterations in growing, healing, and remodeling of maxillofacial tissues. Experimentation is accomplished in humans [209,210], animals [211], or tissue cultures, and histological preparations [212,213].

The compounds are given intravenously to the participants, who are subsequently measured with Geiger counters or subjected to PET/CT scanning as the histochemical markers are taken up from tissues in change. Pictures of the regions of interest are processed and evaluated using a computer-connected gamma camera [210] or special image software [211,214]. The animals receive specially prepared intraperitoneal injections, and the radioactive emission is registered with gamma probes [211].

EMG evaluates the musculature, which functions along with occlusion and is regulated by the nervous system to affect facial proportions. Interestingly, it has been found that in developing rats, the experimental excision of the masseter muscle causes reduction in the angle of the mandible, shortening of the mandible, maxillary asymmetry and, also, specific articular alterations in the temporomandibular joint [215]. Also, Kiliaridis claimed that the masticatory muscles may affect human craniofacial growth when the tension they apply to facial bones exceeds a certain threshold [216].

Regarding the relationship between the EMG and the facial growth patterns at rest, the EMG activity of the anterior masseter, the orbicularis oris, and the anterior digastric muscles was found higher in the vertical growth pattern group. This could be due to the role of these muscles in guiding the mandible in postural position to determine the facial growth pattern. Also, at the maximum central intercuspation, the EMG of the temporalis, the masseter, the buccinator, the orbicularis oris, and the digastric muscles was increased in the horizontal growth pattern group [217]. Tomiyama et al. (2004) reported greater lower lip EMG activity in incompetent patients compared with competent patients [218]. Moreover, surface EMG measurements of the digastric muscle and other facial muscles (zygomaticus major, risorius, superior and inferior orbicularis oris, mentalis, depressor angulioris, and elevator labii superioris) coupled with AI techniques can help estimate static 3D lip shapes [219]. In addition, Zhao et al. (2023), found that the vertical skeletal and the breathing patterns interact and have varying effects on EMG readings [220].

In disagreement with the above-mentioned studies, Cha et al. (2007), Shinkai et al. (2007), and Vianna-Lara et al. (2009) did not correlate facial appearance with orofacial EMG activity [221,222,223]. In addition, Özen & Ceylan (2025) observed only insignificant overall change in the EMG activity of masticatory muscles such as the anterior temporalis and the masseter over a 6-month period following bimaxillary orthognathic surgery in individuals with skeletal Class III malocclusion. Only partial changes in EMG activity were registered during selected functions (chewing, swallowing, and clenching) throughout the trimester of follow-up [224].

## 4. Discussion

### 4.1. Summary of Evidence

In the field of orthodontics, longitudinal growth studies have been organized and run since the beginning of the 20th century with the ambition to examine the normal course of dentofacial development [225]. In the present review, we aimed to include all studies on growth prediction in the context of orthodontics that could be found with the aid of a properly conceived algorithm on the PubMed database. Additional eligible literature was unearthed by manual search and is in the reference lists. Criteria for exclusion were language other than English, and a clear statement of past orthodontic treatment of individuals participating in the sample of study, as such intervention has the potential to alter growth and thus affect the outcome of the method being investigated [226,227,228].

Despite all efforts, some authors did not provide clear details about the subjects that were included in their study. For instance, Fishman (1987) [38], Uysal et al. (2006) [37], and Li et al. (2023) [140] used patient records from private orthodontic practices and from orthodontic departments. Zhou et al. (2021) [132] state that the cephalograms were taken from the oral radiology department. Santiago et al. (2014) [118] accessed cephalometric radiographs that had been taken routinely. Also, Bhatia et al. (1979) [98], Rossouw et al. (1991) [102], and Sato et al. (2001) [55] do not disclose the precise source of their X-rays. Li et al. (2022) [135], Radwan et al. (2022) [137], Seo et al. (2023) [142], and Bulut &Hezenci (2024) [146] do not provide details on any orthodontic interventions applied to their sample. In addition, Mohammad-Rahimi et al. (2022) mentioned that some of the cephalometric radiographs that had been used belonged to patients who might have been treated orthodontically [177]. Kim et al. (2021) [130], Gonca et al. (2024) [147], and Yilmaz & Gonca (2025) [154] used the records of patients that reportedly were in need of orthodontic therapy.

In orthodontics, implant studies are crucial for understanding craniofacial growth and development, particularly regarding skeletal changes due to the spatial remodeling of the facial structures during the period of growth [16,17,99]. Implants, acting as stable reference points, allow researchers to accurately track changes in jaw position and facial dimension patterns during treatment and throughout the patient’s growth period. The most enlightening research on mandibular growth was performed by Björk, who used metallic implants [197]. Björk’s studies have contributed much to the understanding of skeletal and facial growth. However, their application in extreme skeletal patterns requires careful evaluation of the parameters involved [120].

In addition, many animal studies have investigated growth and contributed to the understanding of the biological process and the skeletal remodeling [229,230,231,232,233,234], and how it might be affected by the consistency of diet [235,236,237,238,239]. Growth has also been studied to improve our understanding concerning the relapse of mandibular anterior crowding and how orthodontic appliances advance skeletal change [240,241,242,243].

It seems necessary for the orthodontists to try a kind of growth prediction before starting treatment. Planning where to move the dental units would be unreasonable unless it is envisioned where the bony bases will be at the end of treatment [244]. Predicting growth is important not only in treatment planning, but also in the evaluation of the prognosis throughout the retention and post-retention period [95]. Interventions in cases of developmental deviations and when the treatment plan involves permanent tooth extractions would need an initial assessment of the patient’s growth potential [97,245]. Moreover, cranial proportions, facial dimensions, mandibular size, and occlusal traits may be predicted after considering detrimental habits or abnormal conditions, such as mouth breathing due to impaired airway, frequently appearing in growing individuals [246]. Orthodontic treatment may proceed faster after appropriate time selection to start treatment, in agreement with expected growth, and it is also likely to prove more stable in the long term [32]. Apart from the outcome of treatment, patients’ satisfaction has been reportedly affected by the total duration of treatment, which could be modified by appropriately programming the initiation of treatment [247]. According to Johnston (1968), there seem to be two sources of considerable accuracy in growth prediction, namely the extent to which a distinct structure or a pattern remains stable, and the capability of each orthodontist to predetermine the effects of his own treatment [93]. Currently, it still cannot be verified that the contemporary prediction methods are credible enough to take advantage of available evidence and provide an efficient estimate of long-term changes attributed to growth in an individual. Bhatia et al. (1979) attempted to integrate a variety of cephalometric data in a multivariate statistical model aiming to produce a comprehensive growth prediction model. However, their project was only partially successful [98]. The prognostic accuracy increases when having information on the level of maturity, because this way the normal variability among children of the same age is significantly reduced [31].

A few biological indicators are available for the appraisal of individual skeletal maturity and, consequently, for the detection of the pubertal growth spurt in the mandible [21,248]. Among these, the changes in body height present with the least variability for the assessment of skeletal age throughout the progression of growth, thus being highly reliable as a biological indicator of skeletal maturity [97]. However, a limitation of the method is the need for regularly repeated measurements to construct an individualized curve of growth. Moderate correlations (*r* = 0.42–0.68) have been reported between the skeletal age and some craniofacial measurements, namely the LAFH, the maxillary, and the mandibular dimensions. The mandibular length had a stronger correlation with the upper body size. Sex, and the upper and lower body lengths could be used to determine maxillary length. Skeletal age and body proportions might help in the assessment of the mandibular length [249]. Further, details regarding pubertal growth can also be drawn from reliably formulated, invariant developmental curves. Such graphs are based on longitudinal cephalometric data and their conclusions for any individual should refer to the population means and other statistical values [250].

The correlation coefficients have been found 0.72 (*p* ≤ 0.001) between chronological age and the CVM stages, and 0.79 (*p* ≤ 0.001) between chronological age and maturation as appraised by HWRs. The correlation coefficient between the HWR and the CVM was 0.86 (*p* ≤ 0.001), so the CVM stages are clinically useful to identify skeletal maturity and the timing of the pubertal growth spurt [37]. Jeon et al. (2021) also found that the skeletal maturity assessment from HWRs and the CVM stages in lateral cephalograms had a statistically significant positive correlation in both sexes, and so they can be used effectively for the appraisal of growth by the orthodontist [130]. In agreement, additional research has found similar significant associations in the maturational indices, as evaluated by HWRs and CVM staging [37,111,146]. Moreover, Bulut & Hezenci (2024) reported that the highest correlation was observed in Class III males, while the lowest was found in Class III females and thus, careful consideration should be given to growth assessment. Their findings even suggest that the CVM method can be used for pubertal growth spurt assessment without the need for HWRs [146]. In addition, strong positive association was also demonstrated between chronological age and CVM (*r* = 0.763, *p* ≤ 0.001) [208]. Furthermore, Yamaguchi et al. (2024) provided evidence for particularly using the morphology of the cervical vertebra 4 for predicting the ultimate mandibular length [152].

On the other hand, Hoseini et al. (2016) reported that the level of agreement between the HWR and the CVM was only acceptable in 8- and 9-year-olds of both genders and 10-year-old boys. The level of agreement between the two methods in other age groups was not acceptable. The level of agreement between the two methods was low and, thus, they cannot be used alternatively to estimate the patients’ skeletal age or to predict the peak of growth. This may be due to the effect of different maturation levels, which are influenced by the environment, ethnicity, and gender [174].

According to Cericato et al. (2016), combining dental (third molar development) and skeletal development (CVM) is even more useful for chronological age estimation. Although the above correlations were statistically significant, they should be interpreted with caution. Also, moderate, but acceptable, correlations were registered between dental and skeletal development (*p* < 0.001) [119].

It is important to assess skeletal maturity with reduced exposure to radiation, a reliable and friendly application, which is more suitable for younger patients [100,108]. As an adjunct to standard methods based upon radiographic parameters, the GCF ALP may be a candidate as a non-invasive clinical biomarker for the identification of the pubertal growth spurt in periodontally healthy subjects scheduled for orthodontic treatment. Perinetti et al. (2011) calculated that the GCF-ALP activity increased in the pubertal growth phase as compared to the pre-pubertal and post-pubertal growth phases. The adjusted GCF ALP activity odds ratios for the pre-pubertal and post-pubertal subjects were 0.76 and 0.84, respectively. They found that GCF-ALP activity was a valid, non-invasive biomarker for the identification of the pubertal growth spurt, irrespective of the dentition status [115,122]. Excluding local inflammation [251], variations in GCF-ALP activity might be attributed to serum ALP (systemic factor) and maxillary or mandibular growth (local skeletal factor). In addition, serum ALP activity, a popular biochemical marker indicating osseous turnover, has been reported to increase at puberty and decrease in adulthood [252].

Alhazmi et al. (2019) also agreed on the notion of the salivary ALP activity as a promising diagnostic, non-invasive instrument for predicting pre-pubertal growth. The combination of age and salivary ALP activity may provide the best CVMS prediction compared to other models. They found that combining chronological age and ALP may provide credible CVM stage prediction, suggesting that the use of new tools (biomarkers) and traditional techniques (chronological age) can improve the skeletal maturation assessment. Namely, the salivary ALP values peaked at early puberty, declining afterwards with a significant difference between CVM stages I and II (*p* ≤ 0.001) and between I and V (*p* = 0.004). A significant positive correlation between age and CVM was found [208].

Mandibular growth rate varies throughout development [253], registering a peak during puberty [254]. Among individuals, there is substantial variation in the timing, the intensity, and the duration of the pubertal growth peak [38,255]. The growth spurt periods showed racial differences [37]. Among females, there were no ethnic differences in the pattern or timing of skeletal maturation. In boys, however, skeletal maturity was delayed by 7 months in the black race compared with whites. Therefore, skeletal maturation varies by sex and ethnicity. The delayed maturity of black boys, but not black girls, supports the hypothesis that boys have a greater sensitivity to environmental conditions [256]. To sum up, in African females, the skeletal age is significantly advanced compared to the standards of the Greulich & Pyle atlas. Furthermore, Asian males exhibit a significant delay in bone age between 6 and 9 years of age, to be subsequently considered more advanced at 17 years. Therefore, the classic Greulich & Pyle atlas should be used with caution when applied to populations other than Caucasian origin [257].

Sexual dimorphism in condylar growth has been clearly demonstrated. Male condyles grow at slightly faster rates in childhood and at substantially faster rates during adolescence. Some children exhibit a negative growth velocity around the pre-pubertal minimal growth period and toward the end of adolescence. The condyles follow the general pattern of growth with childhood deceleration, acceleration during adolescence to peak velocity, and rapid deceleration after the peak. Females have less intense rates of condylar growth compared with males, especially during adolescence. They attain peak adolescent velocity approximately 2 years before males, with a substantial individual variation in condylar growth [106].

Nanda (1988) explained that the open- and deep-bite subjects grow differently. Deep-bite subjects are characterized by increased UAFH, while increased LAFH are observed in open-bite persons. Extreme differences appear to be more important than features often attributed to gender differences. However, the posterior face height and the height of the ramus did not differ substantially between the above subjects. The developmental pattern in each facial type is established at a very early age, even before the first permanent molar eruption, long before the adolescent growth spurt. His findings have a connection with the timing of orthodontic treatment, the duration of retention, and the predictability of the emerging occlusal relationship from the mixed dentition up to adulthood [101].

It has been clearly demonstrated that the evaluation of individual skeletal maturity is fundamental in dentofacial orthopedics, as the greatest effects of functional/orthopedic appliances occur when the peak in mandibular growth is included in the treatment period [258]. Chronological age correlates poorly with the growth phases and, in particular, with the onset of the pubertal growth spurt [38,259,260]. In addition, a longitudinal analysis of differences between skeletal and chronological ages showed a wide range of differences during the growth period from 9 to 18 years, despite the differences being small. Nevertheless, the variations highlight the dissimilarities in skeletal maturation among normally growing individuals [261]. The timing of peak mandibular velocity may not be reliably predicted by estimating skeletal age due to high growth variability. So, it might have limited predictive use in an individual patient [19,262]. In addition, the results about the identification of the mandibular growth peak using the CVM method should be interpreted with caution due to reported gender differences [108,263,264] stated that the maturation status of the epiphyses of the male hand bones at 9, 10, or 11 years of age does not predict the timing of peak mandibular growth [112]. Thus, they suggested treating around the average age for greatest rate of growth as an alternative to postponing treatment until the exact occurrence of maximum mandibular lengthening [112].

It is better to use maturational age rather than chronological age when designing orthodontic or surgical treatment. Treatment success is likely to depend on the proper timing, in view of expected maxillomandibular skeletal growth. For many patients, the appropriate timing of intervention might enhance a more desirable outcome in a shorter time [19,113]. Madiraju & Almugla (2024) considered the chronological age as inaccurate to predict skeletal maturation. In addition, the cervical vertebral method of predicting the mandibular growth potential was applicable only to young Saudi males. Chronological age showed a statistically significant strong correlation with CVM age only in young Saudi males [149].

It seems that there is a significant association between hand–wrist skeletal maturation and chronological age. Thus, the HWR can be used for predicting average bone age of an individual because of its simplicity, reliability, and low exposure to radiation [117].

CVM has been advocated as replacement to HWR. It has the advantage that it can be obtained from a usual lateral cephalogram, and extra patient radiation could be prevented. Between methods (CVM and HWR), the correlation values were found moderately high (0.72), but each of them could only predict around 50% of the other method’s skeletal maturation determination. It has been shown that the skeletal level has an impact on the amount of correlation between skeletal maturation as determined by different methods, and this issue should be considered when necessary [111].

The method of CVM has been demonstrated as valid regarding the identification of the pubertal peak in craniofacial growth rate of individuals. The greatest increment in body height takes place at the interval between two morphologic stages in CVM, from stage 3 (when a concavity develops in the inferior border of the third vertebra) to stage 4 (when a concavity develops in the inferior border of the fourth vertebra, and the bodies of all cervical vertebrae become rectangular in shape) in both boys and girls. The peak in statural height during the interval from stage 3 to stage 4 corresponds to the greatest increments in all dimensional and positional mandibular measurements [32,125]. Franchi et al. (2021) suggested combining the CVM with information on the chronological age and the sex to increase reliability in predicting the timing of the pubertal peak in mandibular growth [128].

There is heterogeneity among the studies that use cephalometric analysis to predict future growth. The authors select different cephalometric points and planes to reach conclusions on facial growth. Among the studies, some even suggest associations with distinct anatomical structures such as the frontal/maxillary sinus [102,133,196,265,266,267,268,269,270], the antegonial notch [109,271,272,273], tooth developmental features [114,116,121,123], and the dental location relating to the respective growing pattern [274]. Johnston (1968) suggested that the mandibular, the palatal occlusal plane angles, and the linear size of discrete anatomical structures were not of great predictive value. Instead, the maxillary and mandibular position, their relationship, and area proportions could more frequently be used for useful conclusions [93]. Eventually, the ultimate accuracy of the cephalometric prediction may be limited due to the intrinsic error of the technique itself, not the availability of provided information [93]. Even the introduction of digital methods has not increased the accuracy in identifying cephalometric landmarks to a statistically significant degree. Reliable and accurate, the computer-assisted cephalometric tracing seems to offer a time benefit over the manual approach [242]. Gradually improving AI performance in the field of 3D imaging may eventually contribute in reducing errors attributed to the superimposition of the cephalometric structures [275].

Leslie et al. (1998) indicated that in the general population, the information extracted from a single pre-treatment lateral cephalogram is inadequate for clinically useful predictions pertinent to the extent or direction of future mandibular growth [105]. In addition, Lux et al. (1999) have expressed their disbelief on the value of a single lateral cephalogram to predict future vertical mandibular changes [107].

Facial growth changes are recognized throughout childhood and adolescence. Horizontal mandibular movement does not display an adolescent spurt, whereas vertical movements follow a sigmoidal growth pattern [110]. The anterior movement of menton in girls stabilizes at approximately 12.5 years, but the inferior movement continues. In boys, the horizontal growth of the chin slows slightly, and the vertical movement of the chin increases slightly during adolescence [110].

A computer-based prediction method proved having superior accuracy to the cephalometric analysis needing superimposition, and drawings in a sample with mainly average facial patterns. Normal samples do not usually contain the variety of facial patterns that a clinician may encounter in practice. Interestingly, a small, normal, untreated sample may contain limited developmental deviations, and the computer advantage would be obscured. Repeated comparison of predictions with actual results enabled the computer to predict certain abnormal patterns [96].

In a study comprising 59 males aged 10–19.5 years with an angle Class II division-1 malocclusion, Ruf & Pancherz (1996) suggested that the precision of skeletal maturity assessment by analyzing the maxillary sinus development as depicted on lateral head films was rather high (namely, 85% in the case of l-year prediction interval and 75% when using a 2-year interval). Hence, it was claimed that the method could be used adjunctly with HWRs during the course of orthodontic treatment to highlight an individual’s stage of skeletal development [196]. Rossouw et al. (1991) suggested that the frontal sinus as depicted on a lateral cephalogram may indicate excessive mandibular growth. They reported statistically significant correlations between the size of the frontal sinus on a lateral cephalogram and the condylar length (correlation coefficient *r* = 0.233, *p* < 0.05), the maxillary length (*r* = 0.265, *p* < 0.01), and the mandibular length (*r* = 0.480, *p* < 0.001) [102].

Abate et al. (2022) found that a statistically significant (*p* < 0.05) dimensional increase in the maxillary sinus was correlated with the SNB angle. Namely, the frontal sinus size increased in subjects with greater anterior skeletal dimensions, and with a greater length of the cranial base. Also, an inverse relationship emerged between ANB and the sinus size (*p* = 0.04). The increase in the frontal sinus corresponded to an increase in the length of the skull base. Therefore, it was suggested that in young adults with continuing vertical facial growth, a larger frontal sinus may be associated with future increased vertical growth [133]. Also, according to [265], the maxillary sinus height has a correlation with the basal bone height in the vertical craniofacial pattern. This means that a lower sinus height is seen in the anterior region, increasing in the posterior region, with the horizontal growth pattern exhibiting the least sinus size. The relationship between the sinus height and the facial shape should be related to the frequent nasal obstruction and lymphoid tissue enlargement (adenoids and tonsils) seen in individuals with mouth breathing habits presenting with longer face. There is decreased vertical pneumatization in the anterior sinus region as compared to the posterior because of reduced nasal air circulation in the vertical growth pattern, leading to a more downward increase in posterior maxillary sinus [265]. In addition, Takeda et al. (2025) reported on a positive correlation of facial asymmetry with maxillary sinus length. As facial deviation increased, the maxillary sinus on the non-deviated side tended to grow larger [270].

On the other hand, Asantogrol et al. (2021), Dinç &İçöz (2024), Nimbalkar et al. (2023), and Okşayan et al. (2017) did not observe any statistical differences among skeletal groups regarding the maxillary sinus dimensions. However, Nimbalkar et al. (2023) reported a statistically significant greater maxillary sinus length between Class I and Class III malocclusion coexisting with a vertical skeletal pattern [266,267,268,269].

Kolodziej et al. (2002) investigated the role of the depth of the antegonial notch (an osseous depression on the mandibular lower border, located anteriorly to the angle of the mandible, where the masseter muscle inserts) as predictor of facial growth. Eventually, a statistically, not clinically, significant negative relationship (0.40 ≤ *r ≤* 0.47, *p ≤* 0.05) was revealed between the notch depth in adolescence and the horizontal growth of the maxilla and the mandible up to adulthood. As notch depth decreased, more horizontal jaw growth was observed. Thus, the above concept lacked sufficient justification as a growth predictor [109]. Further, Manabe et al. (2024) showed that the notch area was statistically significantly correlated with the inclination of the ramus (*p* = 0.044, *r* = 0.261) and with the *Y*-axis (*p* = 0.039, *r* = 0.267). This study argued on the potential contribution of the musculature to the notch formation, concurrently with promoting the horizontal growth component [271]. In agreement, Salem et al. (2003) found that the deepening of the notch is associated with a tendency for greater vertical mandibular growth. In addition, both the curve of Spee and the mandibular body length measure decreased when there was an increase in the notch. Namely, there was a statistically significant positive correlation between the antegonial notch and the LAFH (*r* = 0.87, *p* < 0.001). A statistically significant negative correlation was found between the antegonial notch and the mandibular body length (*r* = −0.9, *p* < 0.001). A significant negative statistical relationship existed between the LAFH and the length of the corresponding mandibular bodies, and also between the curve of Spee and the surface area of the respective notch. (*r* = −0.85, *p* < 0.002) [272]. Similarly, Singer et al. (1987) found deep notch subjects having an underdeveloped mandible with a shorter body, less ramus height, and a greater mandibular angle compared to shallow notch individuals. In addition, the mandibular growth direction in deep notch cases, as measured by the facial axis and the MP angle, was more vertically directed. Also, the deep notch subjects had longer total facial height and longer LAFH [273].

According to Solow & Siersbaek-Nielsen (1992), a small craniocervical angle and a backward-inclined upper cervical column has been associated with the face developing more horizontally, the TMJ being displaced backwards, the maxilla lengthening, and greater forward mandibular rotation culminating in a concave facial profile. Contrarily, large craniocervical angle and upright position of the upper cervical column has been connected with vertical facial development, large backward TMJ displacement, reduced maxillary length, reduced mandibular prognathism, and less than average forward mandibular rotation [103].

Associations between the head posture and its structure may be simply explained by functional factors related to the spatial cervical posture. In subjects with vertical cervical posture, a relative anterior positioning of maxillary and mandibular basal structures and the symphysis, in relation to nasion, might be expected. Also, a relative anterior positioning of the mandible in relation to maxilla, a relative increase in the mandibular length in relation to anterior cranial base and the maxilla, an anterior rotation of the mandible, and increased posterior facial height. Decreased anterior total/inferior facial height in relation to the length of the anterior cranial base and increased length of corpus in relation to the height of ramus could also be expected. Opposite craniofacial features should be expected in individuals with forward cervical posture [104].

Issa et al. (2017) and Nayak et al. (2010) agree that the developmental stage of the mandibular permanent canine can be used reliably for the assessment of skeletal maturity [114,116,123]. Moreover, Nawaya & Burhan (2016) noted significant correlations between the permanent first and second mandibular molar crown maturation and the stages of skeletal maturation. Consequently, tooth appearance on panoramic radiographs might be considered as credible predictor of the pubertal growth spurt [121]. However, it is noteworthy, that it has long been argued, that the dental age might prove rather unreliable for appraising maturity [276,277], as dental development may be affected by sex hormones [278]. In addition, Perinetti et al. (2012) found that dental maturation assessment is only useful for diagnosis of the pre-pubertal growth phase [279] and Malik et al. (2012) found that the use of tooth developmental stages should be prescribed only for certain ages [280].

It is not uncommon for lay people to suggest that the youngsters resemble their parents, grandparents, or ancestors [24,281], excluding individuals with known syndromes affecting the craniofacial region [282] and well-known families with extreme facial features that have been connected with inheritance and inbreeding [283,284,285,286]. Data from siblings may be useful for improving prognostic accuracy, depending on the characteristic to be estimated [93].

Research has provided clues to the validity of such claims. In particular, Hunter et al. (1970) found evidence for the inheritance of facial dimensions. The statistical correlations were significantly higher between fathers and offspring, notably regarding the mandible. However, the respective role of the mothers proved to be of lower value apart from the emerging consistent relationship regarding the facial height. Similarities regarding the mandibular dimensions were quite weak [287]. In agreement, Suzuki & Takahama (1991) found a high correlation between the craniofacial form of an offspring and that of his or her parents. Therefore, it is better to use such parental information than average growth curves when the individual growth of a child is to be determined [26,288]. The genetic effects of determining the offspring craniofacial form by the father were equivalent to those by the mother, but the daughters were more affected by parents. The calculated craniofacial form heritability may range from 0.286 to 0.962, with the majority being more than 0.600. Also, the coefficients of correlation between the children and their parents increased with growth, regardless of their orthodontic treatment [289]. Females have more statistically significant variables than the sons. Daughters show similar heritability to both parents, but more variables are highly significant (*p* ≤ 0.001) between daughters and fathers. The sons show stronger heritability to their mothers. The variables with the greatest heritability are those about the position of the lower jaw, the anterior and the posterior face heights, and the cranial base dimensions. Heritability was notably low for the dental variables [289]. On the other hand, Houston & Brown (1980) concluded that family resemblance was not adequately robust for the prediction of facial growth in the individual child. They found that forecasting of facial growth would not improve by considering the facial pattern of other family members. It is likely that a child’s growth pattern might resemble the parents’, and so the orthodontist who possesses cephalometric records of the parents when they were children might be in a better position to forecast future growth of the offspring. Family photographs are more readily available, but rarely would they be suitable for proper analysis. It might be more realistic to investigate whether the growth patterns of older children are worthwhile for predicting the future facial image of their younger relatives. In view of the increasing application of digital instruments, these methods may soon become largely available and easier to use [290].

Santiago et al. (2014) developed a computer software to quantitatively analyze the CVM in order to determine the skeletal age. Their model of prediction including calculations on the vertebral bodies, the chronological age, and gender was considered very satisfactory [118].

AI has been used to assess maxillofacial structures that may have an impact on the shape and function of the oral cavity that the orthodontist aspires to bring into balance [291]. In addition, it has been successfully used for the evaluation of skeletal age by assessing the shape of the cervical vertebrae as they appear on a lateral radiography or by studying the ossification of the small bones on the HWR. Kök et al. (2019) found that ANN could be preferred for determining the CVM stages as it produced the most consistent results compared with other algorithms (k-Nearest Neighbors, logistic regression, support vector machine, random forest, decision tree, and Naive Bayes) [124]. Amasya et al. (2020) reached the same conclusion. Namely, they developed and compared different supervised ML models for the prediction of cervical vertebrae morphology on lateral radiographs. Their study proposed the ANN model because it showed the best performance compared to other tested software (logistic regression, support vector machine, random forest, and decision tree) [126].

Seo et al. (2021) tested different CNN-based DL models (ResNet-18, MobileNet-v2, ResNet-50, ResNet-101, Inception-v3, and Inception-ResNet-v2) to evaluate their performance in determining the stages of CVM on lateral cephalograms. Although all models demonstrated more than 90% accuracy, the Inception-ResNet-v2 had the best relative performance [131]. Zhou et al. (2021) built and trained a CNN model to be used for automatically determining the maturational condition of the cervical vertebrae. Overall, the agreement between the AI and the gold standard was good (correlation coefficient value of up to 98%). In addition, the accuracy of CVM staging was 71%. Hence, AI showed good agreement with human examiners as a useful and reliable means of assessing the CVM [132]. This comes in agreement with the findings of Atici et al. (2022), who investigated DL for fully automated detection and classification of the CVM stages. Their research proposed a custom-designed deep learning CNN with a built-in set of novel tunable directional filters that identify the radiographic edges of the cervical vertebrae. Their suggestion achieved a validation accuracy of 84.63% (statistically significantly decreasing to 80.75% without the directional filters), outperforming other DL models such as the MobileNetV2, the ResNet101 and the Xception [134]. On the contrary, Yilmaz & Gonca (2025) found that different AI regression methods (Ridge, LASSO, and ElasticNet) were clinically insufficient to determine the skeletal age on the basis of the existing maturation of the cervical vertebrae. Allegedly, the results of the study were considered inadequate to decide on the timing of functional intervention or to track the end of growth [154].

Li et al. (2022) evaluated fully automated CVM classification methods based on CNNs (ResNet152, DenseNet161, GoogLeNet, and VGG16). Eventually, ResNet152 proved to be the best model (weighted κ of 0.826 and total accuracy of 67.06%). Nevertheless, all the models proved convenient, fast, and reliable for CVM evaluation [135]. Moreover, Li et al. (2023) created the psc-CVM assessment system, a DL-based, fully automated, high-accuracy CVM assessment software for determining the period of growth. The system performed well with total accuracy of 70.42%. The program exhibited significant consistency with the expert panel and thus, it could be used as a credible aid to identify the developmental maturational stages of the cerebral vertebrae [140]. In addition, Khazaei et al. (2023) developed a DL, CNN for the automatic classification of pubertal growth spurt using CVM. It was based on the ConvNeXtBase-296 architecture achieving an accuracy of up to 93% accuracy [138]. Likewise, the AI software developed by Radwan et al. (2023) showed high reliability and accuracy in determining the pre-pubertal and post-pubertal growth by automatically focusing on the developing cervical vertebral shape [137].

Seo et al. (2023) devised a DL-based approach for the assessment of skeletal age by focusing on the cervical vertebrae on lateral cephalograms of growing children through image segmentation using DeepLabv3+ architecture. The proposed model had average accuracy for the segmentation of cervical vertebrae from lateral cephalogram. The regression model for estimating bone age from segmented cervical vertebrae images yielded average values. Nevertheless, the authors suggested that the method can estimate skeletal age with sufficient accuracy, without the additional need of a HWR [142].

Mohammed et al. (2024) used a CNN-based DL method to predict skeletal growth with the aid of CVM and the mandibular second molar level of calcification as appearing on an ordinary panoramic X-ray. CVM’s accuracy in males was 98%, while females showed high accuracy of second molar calcification. It was concluded that the CNN-mediated classification is adequately accurate to detect the level of maturation, either from CVM or the calcification of the permanent molar [150].

According to Kim et al. (2021), it is possible to predict the maturity of the hand–wrist bones with the aid of cervical vertebral images using an ML method. Additional data on the chronological age and the sex may increase the accuracy in growing individuals. An automated diagnosis of the skeletal maturation may aid as a decision-supporting tool for evaluating the optimal treatment timing for patients [130]. Gonca et al. (2024) found that the application of fractal dimension analysis to HWRs is insufficient to predict maturation stage in growing patients, but it can be improved as method of predicting the rate of growth if combined with the stage of ossification of the sesamoid, the age, and the sex [147].

Jiwa (2020) attempted to implement AI for tracking cephalometric points on serial cephalometric radiographs and carry out a two-year prediction. The results were moderate, but training of the algorithm could potentially improve the technique [127]. Moreover, Myers et al. (2025) managed to predict long-term (8-year period) growth-related changes in skeletal and dental relationships within the craniofacial complex using ML models (LASSO regression, random forest, Support Vector Regression). A clinically acceptable prediction accuracy (within 2 mm or 2°) was achieved for the maxilla to cranial base angle (80%), and the position of the lower incisors (75%) and the angle between the maxilla and mandible (70%). The accuracy was greater for skeletal relationships compared to dental relationships. The pre-pubertal values were recognized as most useful predictive factors concerning post-pubertal measurements [153].

An ML model (LASSO) was formulated by Kim et al. (2023) to predict longitudinal craniofacial growth. Highest prediction accuracy became evident in skeletal linear and angular parameters in 13-year-olds (97.87% and 94.45%, respectively) [139]. Moon et al. (2022) developed a flexible facial growth prediction model incorporating skeletal and soft tissue features. Several predictor variables were incorporated, and several landmarks were predicted [136]. Moon et al. (2024) concluded on the accuracy of a facial growth prediction model using a DL method based on the TabNet deep NN. Its accuracy proved superior to ordinary statistics (lower prediction error by 2.11 mm). Overall, soft-tissue landmarks, mandibular landmarks, and vertical growth showed greater prediction errors than hard-tissue landmarks, landmarks in the maxilla, and growth changes in the horizontal direction, respectively. The multivariate partial least squares regression was accurate in predicting landmarks in the cranial base, which show low variability. However, the AI performed better, particularly for the landmarks in the maxilla and the mandible. Thus, AI may be preferred for growth prediction in case of higher uncertainty [151].

Parrish et al. (2023) created an ML algorithm for predicting the post-pubertal mandibular length and the *Y*-axis in females. The tested ML models could predict post-pubertal mandibular length within 3 mm and *Y*-axis within 1°. Most predictive variables for mandibular length were the mandibular length at previous timepoints, the age, sagittal positions of the maxillary and mandibular skeletal bases, the MP angle, and the anterior and posterior face heights. Most predictive factors for *Y*-axis were the *Y*-axis at previous timepoints, the MP angle, and the sagittal positions of the maxillary and mandibular skeletal bases [141]. In addition, Wood et al. (2023) used ML techniques to predict the post-pubertal mandibular length and the *Y*-axis of growth in males. The accuracy of all the algorithms ranged from 95.80 to 97.64% while predicting post-pubertal mandibular length. When predicting the *Y*-axis of growth, the accuracy ranged from 96.60 to 98.34%. The best predictors for the post-pubertal mandibular length were mandibular and maxillary lengths, and lower face heights at earlier timepoints. The best predictors for the post-pubertal *Y*-axis of growth included the *Y*-axis of growth, lower face height, and MP angle at the earlier timepoints [143].

The male mandibular growth during puberty was studied in males exhibiting Class II malocclusion with a ML model by Zakhar et al. (2023) with the aid of lateral cephalometric radiographs. To predict the degree and the development of the condition, different algorithms were evaluated on a 2- to 4-year forecasting context. Emerging predictive factors for mandibular length were the chronological age, the upper and lower face heights, and the positions and inclinations of the upper and lower incisors. For the *Y*-axis, the most predictive factors were the *Y*-axis at earlier timepoints, SN-MP, SN-Pog, SNB, and SNA. The algorithms of interest successfully predicted the post-pubertal mandibular size within a range of 2.5 mm, and the *y*-axis within 1° [144].

In children with anterior crossbite, an automatic DL, CNN algorithm managed to predict the mandibular growth pattern using cephalometric evidence. Prediction accuracy was good (about 85%) as compared to junior orthodontists (who scored 54.2%). The program mainly focused on the chin, the lower mandibular border, the incisors teeth, the airway, and the condyle [145]. Similarly, it was suggested that a CNN model using hard- and soft-tissue landmarks of the chin area might have the potential for short-term growth prediction (2-year growth interval) in pre-adolescent, Class I patients with longitudinal lateral cephalograms [148].

### 4.2. Strengths and Limitations

Older, well-established techniques and research (dating in the 1960s) were included in the present study along with modern advances in the field of computer technology and digital applications. The authors also chose to describe the research that did not meet the criteria for inclusion in the systematic review, for reasons of thoroughness. Overall, the risk of bias was low for the majority of the included papers, and this increased the reliability of the conclusions of the present study [292]. The search for eligible research involved only the PubMed database. However, more relevant studies were identified and retrieved manually. Reportedly, the performance of current AI-based growth prediction methods may be affected by variability in ethnic background, climatic conditions, and possibly specific dietary habits, highlighting the need for context-specific model adjustments. At last, the methodological variability of the included studies did not allow for meta-analysis.

### 4.3. Recommendations for Future Research

The scientific community should reach a consensus to determine the most appropriate cephalometric landmarks and measurements with the aim of formulating a prediction model based on a single lateral cephalogram. AI can be implemented with the aim to create a comprehensive model including all available information (chronological age, sex, ethnicity, medical records, cephalometric data, appraisal of skeletal age, and dental maturational status) to create an integrated model to predict future growth of the mandible and the face. The proposed models for predicting future facial growth should be evaluated reliably, by organizing randomized control trials.

## 5. Conclusions

Although parental appearance may give a clue regarding the pattern of growth in the offspring, this practice may lead to erroneous conclusions regarding orthodontic treatment-planning, unpleasing outcomes, and questionable stability in the long term. Hence, the assessment of skeletal age is necessary when treating patients that still grow, in agreement with evidence-based practice of orthodontics. The existing methods for predicting future growth are largely inadequate despite the progress made using computer technology. AI can be incorporated in appropriately developed comprehensive models with the aim to produce an integrated tool for long-term prediction of facial proportions.

## Figures and Tables

**Table 1:** Eligibility criteria for the present systematic review.

Domain	Inclusion Criteria	Exclusion Criteria
**Participants**	▪ Healthy individuals or their radiographs	▪ Individuals or their radiographs undergoing orthodontic/orthopedic treatment
**Interventions**	▪ Studying their growth	
**Comparisons**	▪ Different methods	
**Outcomes**	▪ Quantitative or qualitative data regarding growth, [i.e., cephalometric measurements, hand–wrist ossification, cervical vertebrae morphology] measured mainly by radiography [lateral cephalometric radiographs, panoramic radiographs, hand–wrist radiographs etc.]	▪ Photographic assessments regarding growth
**Study design**	▪ Studies describing and comparing methods for growth prediction	▪ Reviews, systematic reviews, and meta-analyses

**Table 2:** Strategy for database search (up to 31 May 2025).

Database	Search Strategy	Hits
**General sources**		
**PubMed** http://www.ncbi.nlm.nih.gov/pubmed	(“growth prediction” OR “skeletal prediction” OR “prediction” OR “growth estimation”) AND (“orthodontics” OR “orthodontic” OR “orthodontic treatment”)	**1047**
**Gray literature sources**		
Reference lists	“growth prediction”	**34**

**Table 3:** Characteristics of the included studies.

Articles	Population	Intervention/Method of Assessment
Johnston, 1968 [93]	Total: 90, 40 F and 50 M	Cephalometric radiographs
2. Björk, 1969 [18]	Total: 100	Cephalometric radiographs—implants
3. Johnston, 1975 [94]	Total: 32, 13 F and 19 M	Cephalometric radiographs
4. Mitchell et al., 1975 [95]	Total: 8, 6 F and 2 M	Subjects selected from earlier implant samples. Cephalometric radiographs
5. Schulhof & Bagha, 1975 [96]	Total: 50	Cephalometric radiographs
6. Grave & Brown, 1976 [97]	Total: 88, 36 F and 52 M	Hand–wrist radiographs
7. Bhatia et al., 1979 [98]	Total: 80, 36 F and 44 M	Cephalometric radiographs
8. Fishman, 1982 [19]	Total: 334, 170 F and 164 M	Hand–wrist radiographs
9. Skieller et al., 1984 [99]	Total: 21, 9 F and 12 M	Cephalometric radiographs—implants
10. Fishman, 1987 [38]	Total: 4.000, 2.225 F and 1.775 M	Hand–wrist radiographs
11. Leite et al., 1987 [100]	Total: 39, 20 F and 19 M	Hand–wrist radiographs
12. Nanda, 1988 [101]	Total: 32, 16 F and 16 M	Cephalometric radiographs
13. Rossouw et al., 1991 [102]	Total: 103	Cephalometric radiographs—frontal sinus
14. Solow & Siersbaek-Nielsen, 1992 [103]	Total: 34, 16 F and 18 M	Cephalometric, cervical vertebrae and hand–wrist radiographs
15. Özbek & Köklü, 1993 [104]	Total: 106, 57 F and 49 M	Cephalometric and cervical vertebrae radiographs
16. Leslie et al., 1998 [105]	Total: 40, 20 F and 20 M	Cephalometric radiographs
17. Buschang et al., 1999 [106]	Total: 221, 108 F and 113 M	Cephalometric radiographs
18. Kocadereli & Telli, 1999 [57]	Total: 40, 20 F and 20 M	Cephalometric radiographs
19. Lux et al., 1999 [107]	Total: 20	Cephalometric radiographs
20. Franchi et al., 2000 [32]	Total: 24, 15 F and 9 M	Cephalometric and cervical vertebrae radiographs
21. Sato et al., 2001 [55]	Total: 44 F	Cephalometric and hand–wrist radiographs
22. Baccetti et al., 2002 [108]	Total: 30, 12 F and 18 M	Cephalometric radiographs for cervical vertebrae maturation evaluation
23. Kolodziej et al., 2002 [109]	Total: 40, 20 F and 20 M	Cephalometric radiographs—antegonial notch depth
24. Chvatal et al., 2005 [110]	Total: 287, 159 F and 128 M	Cephalometric radiographs
25. Flores-Mir et al., 2006 [112]	Total: 79, 52 F and 27 M	Cephalometric radiographs for cervical vertebrae maturation evaluation and hand–wrist radiographs
26. Uysaletal., 2006 [37]	Total: 503, 290 F and 213 M	Cephalometric radiographs for cervical vertebrae maturation evaluation and hand–wrist radiographs
27. Hunter et al., 2007 [112]	Total: 94 M	Cephalometric and hand–wrist radiographs
28. Turchetta et al., 2007 [113]	Total: 50, 26 F and 24 M	Cephalometric radiographs
29. Nayak et al., 2010 [114]	Total: 160, 80 F and 80 M	Periapical radiographs of the mandibular right canines and middle phalanx of third finger
30. Perinetti et al., 2011 [115]	Total: 72, 45 F and 27 M	Gingival crevicular fluid alkaline phosphatase activity and cephalometric radiographs for cervical vertebrae maturation evaluation
31. Hegde et al., 2014 [116]	Total: 160, 80 F and 80 M	Periapical radiographs of the mandibular right canines and middle phalanx of third finger
32. Mohammed et al., 2014 [117]	Total: 330, 165 F and 165 M	Hand–wrist radiographs
33. Santiago et al., 2014 [118]	Total: 236, 120 F and 116 M	Cephalometric radiographs for cervical vertebrae maturation evaluation and hand–wrist radiographs
34. Cericato et al., 2016 [119]	Total: 576, 314 F and 262 M	Cephalometric and panoramic radiographs for cervical vertebrae maturation evaluation
35. Davidovitch et al., 2016 [120]	Total: 395, 258 F and 137 M	Cephalometric radiographs
36. Nawaya & Burhan, 2016 [121]	Total: 262, 137 F and 125 M	Panoramic radiographs for tooth coronal index evaluation and hand–wrist radiographs
37. Perinetti & Contardo, 2016 [122]	Total: 100, 62 F and 38 M	Gingival crevicular fluid alkaline phosphatase activity and cephalometric radiographs for cervical vertebrae maturation evaluation
38. Issa et al., 2017 [123]	Total: 292, 142 F and 150 M	Panoramic radiographs for open apex measurements of the lower teeth and hand–wrist radiographs
39. Kök et al., 2019 [124]	Total: 300	Cephalometric radiographs for cervical vertebrae maturation evaluation
40. Montasser, 2019 [125]	Total: 26, 12 F and 14 M	Cephalometric and cervical vertebrae radiographs
41. Amasya et al., 2020 [126]	Total: 647, 343 F and 304 M	Cervical vertebral maturation assessment on lateral cephalometric radiographs using artificial intelligence
42. Jiwa, 2020 [127]	Total: 101, 49 F and 52 M	Cephalometric radiographs
43. Franchi et al., 2021 [128]	Total: 50, 29 F and 21 M	Cephalometric radiographs for cervical vertebrae maturation evaluation
44. Jeon et al., 2021 [129]	Total: 1.017, 614 F and 403 M	Cephalometric radiographs for cervical vertebrae maturation evaluation and hand–wrist radiographs
45. Kim et al., 2021 [130]	Total: 455, 272 F and 227 M	Cephalometric radiographs for cervical vertebrae maturation evaluation and hand–wrist radiographs
46. Seo et al., 2021 [131]	Total: 600	Deep learning models for cervical vertebral maturation stage classification on lateral cephalometric radiographs
47. Zhou et al., 2021 [132]	Total: 1.080, 605 F and 475 M	Artificial intelligence for automatic evaluation of cervical vertebral maturation status
48. Abate et al., 2022 [133]	Total: 80, 40 F and 40 M	Cone Beam Computed Tomography scans for volumetric morphological assessment of the frontal sinus
49. Atici et al., 2022 [134]	Total: 1.018	Deep learning models for cervical vertebral maturation stage classification on lateral cephalometric radiographs
50. Li et al., 2022 [135]	Total: 6.079, 3.503 F and 2.576 M	Convolutional neural networks for cervical vertebral maturation stage classification on lateral cephalometric radiographs
51. Moon et al., 2022 [136]	Total: 303, 166 F and 137 M	Cephalometric radiographs and partial least squares algorithm
52. Radwan et al., 2022 [137]	Total: 1.501	Convolutional neural networks for cervical vertebral maturation stage classification on lateral cephalometric radiographs
53. Khazaei et al., 2023 [138]	Total:1.846, 1.163 F and 683 M	Convolutional neural networks for cervical vertebral maturation stage classification on lateral cephalometric radiographs
54. Kim et al., 2023 [139]	Total: 59, 32 F and 27 M	Cephalometric radiographs and machine learning models
55. Li et al., 2023 [140]	Total:10.200	Deep learning models for cervical vertebral maturation stage classification on lateral cephalometric radiographs
56. Parrish et al., 2023 [141]	Total: 176 F	Cephalometric radiographs
57. Seo et al., 2023 [142]	Total: 900, 444 F and 456 M	Deep learning models for cervical vertebral maturation stage classification on lateral cephalometric radiographs
58. Wood et al., 2023 [143]	Total: 163 M	Cephalometric radiographs and machine learning techniques
59. Zakhar et al., 2023 [144]	Total: 123 M	Cephalometric radiographs and machine learning techniques
60. Zhang et al., 2023 [145]	Total: 296, 154 F and 142 M	Cephalometric radiographs and deep learning model
61. Bulut &Hezenci, 2024 [146]	Total: 1000, 526 F and 474 M	Cephalometric radiographs for cervical vertebrae maturation evaluation and hand–wrist radiographs
62. Gonca et al., 2024 [147]	Total: 1.067	Hand–wrist radiographs
63. Larkin et al., 2024 [148]	Total: 198, 80 F and 80 M	Cephalometric radiographs and convolutional neural network algorithm
64. Madiraju &Almugla, 2024 [149]	Total: 80, 40 F and 40 M	Cephalometric and cervical vertebrae radiographs
65. Mohammed et al., 2024 [150]	Total: 1200 cephalometric and 1200 panoramicradiographs	Cephalometric radiographs for cervical vertebrae maturation evaluation and the mandibular right second molar
66. Moon et al., 2024 [151]	Total: 410, 236 F and 174 M	Cephalometric radiographs and partial least squares algorithm
67. Yamaguchi et al., 2024 [152]	Total: 44 F	Cephalometric and cervical vertebrae radiographs
68. Myers et al., 2025 [153]	Total: 301	Cephalometric radiographs and machine learning techniques
69. Yilmaz & Gonca, 2025 [154]	Total: 794, 465 F and 329 M	Cephalometric radiographs for cervical vertebrae maturation evaluation and hand–wrist radiographs

**Table 4:** Summary of risk of bias assessment.

	Signaling Questions								
Study	1	2	3	4	5	6	7	8	Summary
Johnston, 1968 [93]	Yes	Yes	Unclear	Yes	No	No	Yes	Yes	Low
2. Björk, 1969 [18]	Yes	Yes	Unclear	Yes	No	No	Yes	Yes	Low
3. Johnston, 1975 [94]	Yes	Yes	Unclear	Yes	No	No	Yes	Yes	Low
4. Mitchell et al., 1975 [95]	Yes	Yes	Unclear	Yes	No	No	Yes	Yes	Low
5. Schulhof & Bagha, 1975 [96]	Yes	Yes	Unclear	Yes	No	No	Yes	Yes	Low
6. Grave & Brown, 1976 [97]	Yes	Yes	Unclear	Yes	No	No	Yes	Yes	Low
7. Bhatia et al., 1979 [98]	Yes	Yes	Unclear	Yes	No	No	Yes	Yes	Low
8. Fishman, 1982 [19]	Yes	Yes	Unclear	Yes	No	No	Yes	Yes	Low
9. Skieller et al., 1984 [99]	Yes	Yes	Unclear	Yes	No	No	Yes	Yes	Low
10. Fishman, 1987 [38]	Yes	Yes	Unclear	Yes	No	No	Yes	Yes	Low
11. Leite et al., 1987 [100]	Yes	Yes	Unclear	Yes	No	No	Yes	Yes	Low
12. Nanda, 1988 [101]	Yes	Yes	Unclear	Yes	No	No	Yes	Yes	Low
13. Rossouw et al., 1991 [102]	Yes	Yes	Unclear	Yes	No	No	Yes	Yes	Low
14. Solow &Siersbaek-Nielsen, 1992 [103]	Yes	Yes	Unclear	Yes	No	No	Yes	Yes	Low
15. Özbek &Köklü, 1993 [104]	Yes	Yes	Unclear	Yes	No	No	Yes	Yes	Low
16. Leslie et al., 1998 [105]	Yes	Yes	Unclear	Yes	No	No	Yes	Yes	Low
17. Buschang et al., 1999 [106]	Yes	Yes	Unclear	Yes	No	No	Yes	Yes	Low
18. Kocadereli& Telli, 1999 [57]	Yes	Yes	Unclear	Yes	No	No	Yes	Yes	Low
19. Lux et al., 1999 [107]	Yes	Yes	Unclear	Yes	No	No	Yes	Yes	Low
20. Franchi et al., 2000 [32]	Yes	Yes	Unclear	Yes	No	No	Yes	Yes	Low
21. Sato et al., 2001 [55]	Yes	Yes	Unclear	Yes	No	No	Yes	Yes	Low
22. Baccetti et al., 2002 [108]	Yes	Yes	Unclear	Yes	No	No	Yes	Yes	Low
23. Kolodziej et al., 2002 [109]	Yes	Yes	Unclear	Yes	No	No	Yes	Yes	Low
24. Chvatal et al., 2005 [110]	Yes	Yes	Unclear	Yes	No	No	Yes	Yes	Low
25. Flores-Mir et al., 2006 [111]	Yes	Yes	Unclear	Yes	No	No	Yes	Yes	Low
26. Uysal et al., 2006 [37]	Yes	Yes	Unclear	Yes	No	No	Yes	Yes	Low
27. Hunter et al., 2007 [112]	Yes	Yes	Unclear	Yes	No	No	Yes	Yes	Low
28. Turchetta et al., 2007 [113]	Yes	Yes	Unclear	Yes	No	No	Yes	Yes	Low
29. Nayak et al., 2010 [114]	Yes	Yes	Unclear	Yes	No	No	Yes	Yes	Low
30. Perinetti et al., 2011 [115]	Yes	Yes	Unclear	Yes	No	No	Yes	Yes	Low
31. Hegde et al., 2014 [116]	Yes	Yes	Unclear	Yes	No	No	Yes	Yes	Low
32. Mohammed et al., 2014 [117]	Yes	Yes	Unclear	Yes	No	No	Yes	Yes	Low
33. Santiago et al., 2014 [118]	Yes	Yes	Unclear	Yes	No	No	Yes	Yes	Low
34. Cericato et al., 2016 [119]	Yes	Yes	Unclear	Yes	No	No	Yes	Yes	Low
35. Davidovitch et al., 2016 [120]	Yes	Yes	Unclear	Yes	No	No	Yes	Yes	Low
36. Nawaya et al., 2016 [121]	Yes	Yes	Unclear	Yes	No	No	Yes	Yes	Low
37. Perinetti& Contardo, 2016 [122]	Yes	Yes	Unclear	Yes	No	No	Yes	Yes	Low
38. Issa et al., 2017 [123]	Yes	Yes	Unclear	Yes	No	No	Yes	Yes	Low
39. Kök et al., 2019 [124]	Yes	Yes	Unclear	Yes	No	No	Yes	Yes	High
40. Montasser, 2019 [125]	Yes	Yes	Unclear	Yes	No	No	Yes	Yes	Low
41. Amasya et al., 2020 [126]	Yes	Yes	Unclear	Yes	Yes	No	Yes	Yes	Low
42. Jiwa, 2020 [127]	Yes	Yes	Unclear	Yes	No	No	Yes	Yes	Low
43. Franchi et al., 2021 [128]	Yes	Yes	Unclear	Yes	No	No	Yes	Yes	Low
44. Jeon et al., 2021 [129]	Yes	Yes	Unclear	Yes	No	No	Yes	Yes	Low
45. Kim et al., 2021 [130]	No	No	Unclear	Yes	Yes	No	Yes	Yes	High
46. Seo et al., 2021 [131]	Yes	No	Unclear	Yes	Yes	No	Yes	Yes	High
47. Zhou et al., 2021 [132]	Yes	Yes	Unclear	Yes	Yes	No	Yes	Yes	Low
48. Abate et al., 2022 [133]	Yes	Yes	Unclear	Yes	No	No	Yes	Yes	Low
49. Atici et al., 2022 [134]	No	No	Unclear	Yes	Yes	No	Yes	Yes	High
50. Li et al., 2022 [135]	Yes	Yes	Unclear	Yes	No	No	Yes	Yes	Low
51. Moon et al., 2022 [136]	Yes	Yes	Unclear	Yes	No	No	Yes	Yes	Low
52. Radwan et al., 2022 [137]	Yes	Yes	Unclear	Yes	No	No	Yes	Yes	Low
53. Khazaei et al., 2023 [138]	Yes	Yes	Unclear	Yes	No	No	Yes	Yes	Low
54. Kim et al., 2023 [139]	Yes	Yes	Unclear	Yes	No	No	Yes	Yes	Low
55. Li et al., 2023 [140]	Yes	Yes	Unclear	Yes	No	No	Yes	Yes	Low
56. Parrish et al., 2023 [141]	Yes	Yes	Unclear	Yes	No	No	Yes	Yes	Low
57. Seo et al., 2023 [142]	Yes	Yes	Unclear	Yes	No	No	Yes	Yes	Low
58. Wood et al., 2023 [143]	Yes	Yes	Unclear	Yes	No	No	Yes	Yes	Low
59. Zakhar et al., 2023 [144]	Yes	Yes	Unclear	Yes	No	No	Yes	Yes	Low
60. Zhang et al., 2023 [145]	Yes	Yes	Unclear	Yes	No	No	Yes	Yes	Low
61. Bulut &Hezenci, 2024 [146]	Yes	Yes	Unclear	Yes	No	No	Yes	Yes	Low
62. Gonca et al., 2024 [147]	Yes	Yes	Unclear	Yes	No	No	Yes	Yes	Low
63. Larkin et al., 2024 [148]	Yes	Yes	Unclear	Yes	No	No	Yes	Yes	Low
64. Madiraju &Almugla, 2024 [149]	Yes	Yes	Unclear	Yes	No	No	Yes	Yes	Low
65. Mohammed et al., 2024 [150]	Yes	Yes	Unclear	Yes	No	No	Yes	Yes	Low
66. Moon et al., 2024 [151]	Yes	Yes	Unclear	Yes	No	No	Yes	Yes	Low
67. Yamaguchi et al., 2024 [152]	Yes	Yes	Unclear	Yes	No	No	Yes	Yes	Low
68. Myers et al., 2025 [153]	Yes	Yes	Unclear	Yes	No	No	Yes	Yes	Low
69. Yilmaz & Gonca, 2025 [154]	Yes	Yes	Unclear	Yes	No	No	Yes	Yes	Low

1: Were the criteria for inclusion in the sample clearly defined?; 2: Were the study subjects and the setting described in detail?; 3: Was the exposure measured in a valid and reliable way?; 4: Were objective, standard criteria used for measurement of the condition?; 5: Were confounding factors identified?; 6: Were strategies to deal with confounding factor stated?; 7: Were the outcomes measured in a valid and reliable way?; 8: Was appropriate statistical analysis used?

## Abbreviations

AI	artificial intelligence
ALP	alkaline phosphatase
ANN	artificial neural network-based
CNN	convolutional neural network
CVM	cervical vertebral maturation
DL	deep learning
EMG	electromyography
FD	fractal dimension
FH	Frankfurt horizontal
GCF	gingival crevicular fluid
HWR	hand–wrist radiograph
LAFH	lower anterior facial height
ML	machine learning
MP	mandibular plane
NN	neural network
PLS	partial least square
SD	standard deviation
TMJ	temporomandibular joint
TW	Tanner–Whitehouse
UAFH	upper anterior facial height
